# Rheological, Microstructural and Thermal Properties of Magnetic Poly(Ethylene Oxide)/Iron Oxide Nanocomposite Hydrogels Synthesized Using a One-Step Gamma-Irradiation Method

**DOI:** 10.3390/nano10091823

**Published:** 2020-09-12

**Authors:** Ivan Marić, Nataša Šijaković Vujičić, Anđela Pustak, Marijan Gotić, Goran Štefanić, Jean-Marc Grenèche, Goran Dražić, Tanja Jurkin

**Affiliations:** 1 Radiation Chemistry and Dosimetry Laboratory, Division of Materials Chemistry, Ruđer Bošković Institute, Bijenička Cesta 54, 10000 Zagreb, Croatia; imaric@irb.hr (I.M.); apustak@irb.hr (A.P.); 2 Laboratory for Supramolecular Chemistry, Division of Organic Chemistry and Biochemistry, Ruđer Bošković Institute, Bijenička Cesta 54, 10000 Zagreb, Croatia; Natasa.Sijakovic-Vujicic@irb.hr; 3 Laboratory for Molecular Physics and Synthesis of New Materials, Division of Materials Physics, Ruđer Bošković Institute, Bijenička Cesta 54, 10000 Zagreb, Croatia; gotic@irb.hr (M.G.); Goran.Stefanic@irb.hr (G.Š.); 4 Institut des Molécules et Matériaux du Mans (IMMM UMR CNRS 6283), Le Mans Université, Avenue Olivier Messiaen, F-72085 Le Mans CEDEX 9, France; jean-marc.greneche@univ-lemans.fr; 5 Department of Materials Chemistry, National Institute of Chemistry, Hajdrihova 19, SI-1001 Ljubljana, Slovenia; goran.drazic@ki.si

**Keywords:** magnetic hydrogel, gamma-irradiation, poly(ethylene oxide), magnetite, rheological properties, thermal properties, ^57^Fe Mössbauer spectrometry, XRD, SEM, Fe(II) determination

## Abstract

Magnetic polymer gels are a new promising class of nanocomposite gels. In this work, magnetic PEO/iron oxide nanocomposite hydrogels were synthesized using the one-step γ-irradiation method starting from poly(ethylene oxide) (PEO) and iron(III) precursor alkaline aqueous suspensions followed by simultaneous crosslinking of PEO chains and reduction of Fe(III) precursor. γ-irradiation dose and concentrations of Fe^3+^, 2-propanol and PEO in the initial suspensions were varied and optimized. With 2-propanol and at high doses magnetic gels with embedded magnetite nanoparticles were obtained, as confirmed by XRD, SEM and Mössbauer spectrometry. The quantitative determination of γ-irradiation generated Fe^2+^ was performed using the 1,10-phenanthroline method. The maximal Fe^2+^ molar fraction of 0.55 was achieved at 300 kGy, pH = 12 and initial 5% of Fe^3+^. The DSC and rheological measurements confirmed the formation of a well-structured network. The thermal and rheological properties of gels depended on the dose, PEO concentration and initial Fe^3+^ content (amount of nanoparticles synthesized inside gels). More amorphous and stronger gels were formed at higher dose and higher nanoparticle content. The properties of synthesized gels were determined by the presence of magnetic iron oxide nanoparticles, which acted as reinforcing agents and additional crosslinkers of PEO chains thus facilitating the one-step gel formation.

## 1. Introduction

Magnetic polymer gels (ferrogels) are a new and promising class of nanocomposite hydrogels that have the potential to be used as effective absorbents of toxic ions in water, protein immobilization, separation, in soft actuators such as artificial muscles, in tissue engineering, drug delivery and hyperthermia applications [1,2,3,4,5,6,7]. Ferrogels combine the elastic properties and the defined structure of gels with the magnetic properties of magnetic nanoparticles (usually magnetite or maghemite) and respond quickly to the external magnetic field. Apart from their unique magnetic properties, nanocomposite gel scaffolds with embedded magnetite/maghemite nanoparticles have exhibited superior mechanical, rheological and electrical properties compared to scaffold gels without nanoparticle reinforcement, better biocompatibility, low cytotoxicity, and demonstrated antibacterial properties [1,2,3,4]. Furthermore, iron oxide nanoparticles have been shown to promote osteogenic differentiation of stem cells [8,9,10] and can provide the transduction of the dynamic mechanical stimulation required for bone formation [11].

Nanocomposite gels can be synthesized by a variety of methods, including the radiolytic method. γ-irradiation (radiolytic method) has the advantage of a pure and homogeneous initiation of the polymer crosslinking reaction and reduction of metal cations, as well as the sterility of the final product. Typically, nanocomposite gels are synthesized by two-step methods: (i) γ-irradiation induced crosslinking of the polymer in solution in the presence of pre-prepared nanoparticles (NPs) [12,13] or (ii) in situ γ-irradiation synthesis of nanoparticles within the already prepared polymer gel [14,15,16,17]. Of particular interest is the one-step γ-irradiation synthesis of nanocomposite gels. However, the one-step synthesis has the advantage of simultaneous crosslinking of polymer chains with the formation of network and reduction of metal salts and the formation of NPs. The one-step synthesis is faster and simpler and results in a small NPs size and narrow size distribution as well as homogeneous distribution of NPs throughout the polymer matrix. On the other hand, the one-step synthesis of nanocomposite gels has been poorly studied, as it is difficult to find favorable conditions for both nanoparticle synthesis and polymer crosslinking.

The γ-irradiation method is highly suitable for the synthesis of NPs of controlled size and shape in a solution and in heterogeneous media such as hydrogels. It is also suitable for forming the three-dimensional polymer network. However, the studies are mostly oriented toward the synthesis of metal NPs and gels containing metal NPs. The radiolytic synthesis of iron oxide nanoparticles and nanocomposite gels is rarely studied [18,19,20,21,22,23,24,25,26,27,28]. One of the reasons for this is a very complex iron oxide chemistry, which generates numerous iron oxide and oxyhydroxide polymorphs. Another difficulty is the high susceptibility of magnetic iron oxide NPs to (re)oxidation of ferrous ions (Fe^2+^) to ferric ions (Fe^3+^) under atmospheric conditions, especially when they are in the nano-size range. The main principle of formation of metal and metal oxide nanoparticles by gamma-irradiation of an aqueous solution of a metal salt or within a hydrogel is the reduction of metal cations (M^n+^) by hydrated electrons ($e_{aq}^{-}$) (*E*^o^(H_2_O/$e_{aq}^{-}$) = −2.87 V_SHE_) and proton radicals (H**·**) (*E*^o^(H^+^/H^•^) = −2.30 V_SHE_) which are strong reducing agents formed on water radiolysis [29],

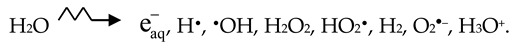
(1)

These reducing species can easily reduce metal cations with a more positive standard potential to a lower oxidation state or zero-valence state, such as ferric ions to ferrous (*E*^o^(Fe^3+^/Fe^2+^) = +0.77 V) or even Fe^0^ ((*E*^o^(Fe^3+^/Fe^0^) = −0.04 V; *E*^o^(Fe^2+^/Fe^0^) = −0.44 V) [18,26]. Due to the high energy and penetration of γ-radiation, strong reducing species are formed homogeneously throughout the system, resulting in homogeneous NPs nuclei formation. On the other hand, hydroxyl radicals (^•^OH), as well as HO_2_^•^ and O_2_^•−^ radicals formed on water radiolysis, are strong oxidizing species (V_NHE_, *E*^o^(^•^OH/H_2_O) = +2.34 V_NHE_). In order to ensure strong reducing conditions, irradiation is carried out in deoxygenated solutions and with the addition of scavengers of hydroxyl radicals, such as 2-propanol. The formed 2-propanol radicals can also reduce metal cations (*E*^o^((CH_3_)_2_CO/(CH_3_)_2_C^•^OH = −1.8 V_NHE_).

In our previous works, we synthesized different magnetic iron oxide NPs using γ-irradiation in the presence of various polymers and surfactants (polyvinylpyrrolidone (PVP), cetyltrimethylammonium bromide (CTAB), DEAE-dextran, dextran sulfate) [23,24,25] and within microemulsion droplets [20,21] in order to control the stability of the synthesized magnetic suspensions and the size and morphology of the nanoparticles. The aim of this work was to explore the ability of γ-irradiation technique for the one-step synthesis of magnetic poly(ethylene oxide)/Fe-oxide (PEO/Fe-oxide) nanocomposite hydrogels. PEO was selected because it is a semicrystalline, hydrophilic and biocompatible polymer with numerous applications in pharmacy and biomedicine. For instance, it is used as wound dressings and hydrogels for active substance release. Upon γ-irradiation of PEO aqueous solutions, PEO easily crosslinks and forms macroscopic “wall-to-wall” hydrogels [12,30,31,32,33]. In this work, we optimized the experimental conditions and synthesized magnetic PEO/iron oxide nanocomposite hydrogels in one step starting from an alkaline aqueous suspension of PEO and Fe^3+^ precursor. In addition, the influence of γ-irradiation dose and concentrations of Fe^3+^ precursor and PEO on the microstructural, thermal and rheological properties of such one-step synthesized magnetic nanocomposite gels was studied.

## 2. Materials and Methods

### 2.1. Chemicals for the Synthesis

All chemicals were of analytical purity and used as received. Iron(III) chloride hexahydrate (FeCl_3_·6H_2_O) (puriss. p.a., Reag. Ph. Eur., ≥99%) produced by Sigma-Aldrich, St. Louis, MO, USA; sodium hydroxide (anhydrous, free-flowing, pellets, ACS reagent, ≥97%) by Sigma-Aldrich, St. Louis, MO, USA/Honeywell, Muskegon, MI, USA; 2-propanol (CROMASOLV, for HPLC, ≥99.9%) by Sigma-Aldrich, St. Louis, MO, USA/Honeywell, Muskegon, MI, USA; poly(ethylene oxide) (PEO) of viscosity average molecular weight (M_v_) 400,000 by Sigma-Aldrich, St. Louis, MO, USA and Mili-Q deionized water were used.

### 2.2. Synthesis of Samples

**Figure 1:**
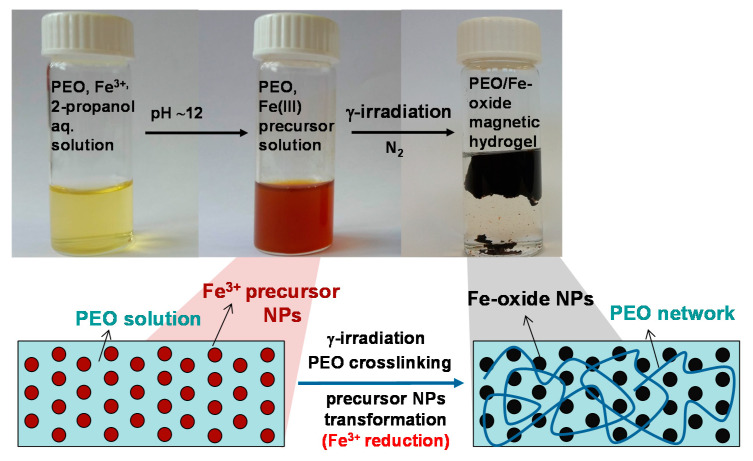
Schematic presentation of the synthesis procedure of magnetic poly(ethylene oxide) (PEO)/iron oxide nanocomposite hydrogels.

Iron(III) chloride salt was used for the synthesis of iron oxide nanoparticles. PEO/iron(III) precursor suspensions were prepared by firstly dissolving PEO powder to prepare 1.85 wt% aqueous solutions, followed by the addition of 2 M FeCl_3_ aqueous solution to the final concentrations of initial Fe^3+^ ions in solutions being 0.35 × 10^−2^ M, 1.75 × 10^−2^ M, and 7 × 10^−2^ M. These Fe^3+^ concentrations in solutions correspond to mass percentages of Fe^3+^ of 1, 5, 20 wt% relative to total PEO and Fe^3+^ mass, respectively. In addition, one batch of precursor solutions wasprepared from 4 wt% PEO aqueous solutions with the same concentrations of Fe^3+^ salt. The solutions were irradiated with or without 2-propanol. The final concentration of 2-propanol in solutions was 0.2 M. In addition, few solutions were prepared containing four times more 2-propanol (0.8 M). The pH of suspensions was adjusted to pH = 11.5–12 with 2 M NaOH aqueous solution. The prepared precursor suspensions were bubbled with nitrogen through rubber septa for 30 min in order to remove dissolved oxygen before γ-irradiation. γ-irradiation of deoxygenated suspensions in septum-closed glass vials was performed at room temperature in a ^60^Co γ-irradiation facility located in the Radiation Chemistry and Dosimetry Laboratory at the Ruđer Bošković Institute. The suspensions were irradiated to doses of 50, 130 and 300 kGy and at a dose rate of ~27 kGy h^−1^. Irradiation of PEO/Fe(III) precursor suspensions resulted in the formation of gels or suspensions. The schematic presentation of the synthesis procedure of magnetic PEO/iron oxide nanocomposite hydrogels is given in Figure 1.

### 2.3. Characterization of Samples

Synthesized samples were characterized as as-synthesized gels (rheological measurements), as dried gels (DSC, XRD, Mössbauer, SEM) or as suspensions/isolated precipitates (XRD, Mössbauer). Precipitates were isolated from suspensions by centrifugation, followed by washing with ethanol. *Scanspeed* 2236R high-speed centrifuge was used. The obtained gels and isolated precipitates were dried in vacuum at room temperature, and then characterized.

The morphology of samples was investigated using a probe Cs-corrected cold field-emission Scanning Transmission Electron Microscope (STEM, model ARM 200 CF), and the thermal field emission scanning electron microscope (FE SEM, model JSM-7000F) manufactured by JEOL Ltd., Tokyo, Japan, FE SEM was linked to the EDS/INCA 350 (energy-dispersive X-ray analyzer) manufactured by Oxford Instruments Ltd., Abingdon, UK.

X-ray powder diffraction (XRD) patterns were recorded at room temperature using an APD 2000 X-ray powder diffractometer (Cu*K*α radiation, graphite monochromator, NaI–Tl detector) manufactured by *ItalStructures*, Riva Del Garda, Italy. The XRD patterns were recorded over the 15–80° 2*θ* range with a 2*θ* step of 0.05–0.025° and a counting time per step of 15–80 s.

^57^Fe Mössbauer spectra were recorded at 20 °C in the transmission mode using a standard instrumental configuration by WissEl GmbH, Mömbris-Hohl, Germany. The ^57^Co in the rhodium matrix was used as a Mössbauer source. The spectrometer was calibrated at 20 °C using the standard α-Fe foil spectrum. The velocity scale and all the data refer to the metallic α-Fe absorber at 20 °C. The experimentally observed Mössbauer spectra were fitted using the MossWinn program. Additionally, ^57^Fe Mössbauer spectra were recorded at 77 K using a conventional constant acceleration transmission spectrometer with a ^57^Co(Rh) source and a bath cryostat. The spectra obtained at 77 K were fitted using the MOSFIT program (Teillet, J.; Varret, F. unpublished MOSFIT program, Université du Maine) and an α-Fe absorber was used as a calibration sample.

Differential scanning calorimetry (DSC) thermograms were recorded using PerkinElmer, Waltham, MA, USA Diamond DSC calorimeter, calibrated with In and Zn standards and operating in a dynamic mode. Samples of dried gel (5–10 mg) were sealed into Al pans. Two heating and cooling cycles at temperatures ranging from −40 °C to 100 °C in an extra pure nitrogen environment were performed for each sample at a rate of 10 °C min^−1^. The first heating cycle was performed in a range 22 °C to 100 °C. For each synthesized gel three specimens were recorded. The temperatures and enthalpies of melting and crystallization were determined from the second heating and first cooling cycles, and their averages are presented.

The mechanical properties of gels are described using oscillatory rheology. The storage (*G*′) and loss (*G*″) moduli of the nanocomposite gels were determined with a mechanical spectrometer (Anton Paar MCR 302, Stuttgart, Germany), using a steel plate−plate geometry (PP25, Anton Paar, Graz, Austria) equipped with a true-gap system and the data were collected using RheoCompass software. The sample temperature was controlled through a Peltier temperature control located on the base of the geometry and with a Peltier-controlled hood (H-PTD 200). A piece of a gel sample (1 mm thick slice) was placed on the base plate of the rheometer, and the plate was set using the true-gap function of the software. Thus, after 5 min at 25 °C, the *G*′ and *G*″ moduli were measured always within the linear viscoelastic region (LVR). After 5 min at 25 °C, the yield stress of the gels was determined by applying a strain (γ) sweep between 0.01% and 100%. Rheological properties of the gel material are independent of strain up to yield strain, and beyond yield strain the rheological behavior is nonlinear. Three interval thixotropy test is a standard test which allows tracking of material response resulting from stepwise changes in shear strain making it the most appropriate method for structure recovery tests. In the thixotropic experiments, rheological measurements were conducted on gels at 25 °C under initial conditions at which they were in their linear viscoelastic regimes (a strain of 0.1% and angular frequency of 5 rad/s) for 680 s to establish baseline values for *G*′ and *G*″. In studies with gels viscoelastic recovery was observed after the cessation of destructive strain. Frequency sweeps (0.05–100 rad/s) were subsequently performed at 25 °C at a strain value within LVR to investigate the time-dependent deformation behavior of gels.

### 2.4. Quantitative Determination of Fe^2+^ Using 1,10-Phenanthroline UV-Vis Spectrophotometric Method

#### 2.4.1. Chemicals for Spectrophotometric Determination

All chemicals were of analytical purity and used as received. Sodium acetate (Merck, Kenilworth, NJ, USA anhydrous for analysis, EMSURE, ACS, Reag. Ph. Eur.), acetic acid (Honeywell, Muskegon, MI, USA puriss.p.a., ACS Reagent, Reag. Ph. Eur., ≥99.8%), L-ascorbic acid (Sigma-Aldrich, St. Louis, MO, USA BioXtra, crystalline, ≥99.0%), 1,10-phenanthroline monohydrate (Sigma-Aldrich, St. Louis, MO, USA for the spectrophotometric determination, ≥99.0%), and hydrochloric acid (Fluka (Honeywell), Muskegon, MI, USA for trace analysis, fuming, ≥37%) were used.

#### 2.4.2. Spectrophotometric Determination of Fe^2+^

The amount of Fe^2+^ generated upon γ-irradiation was determined using the 1,10-phenanthroline method [34,35].

Immediately after irradiation the samples were acidified (pH ≤ 1) by the addition of concentrated hydrochloric acid (~2.5 vol%) using a syringe through rubber septa. At such low pH, formed iron oxide nanoparticles are dissolved and all Fe^2+^ formed upon γ-irradiation is preserved from oxidation when the vial is opened. The detailed procedure for the determination of Fe^2+^ and total iron in such acidified solutions is given in our recently published paper [35].

## 3. Results and Discussion

### 3.1. Microstructural Characterization of Gels (and Suspensions)

**Figure 2:**
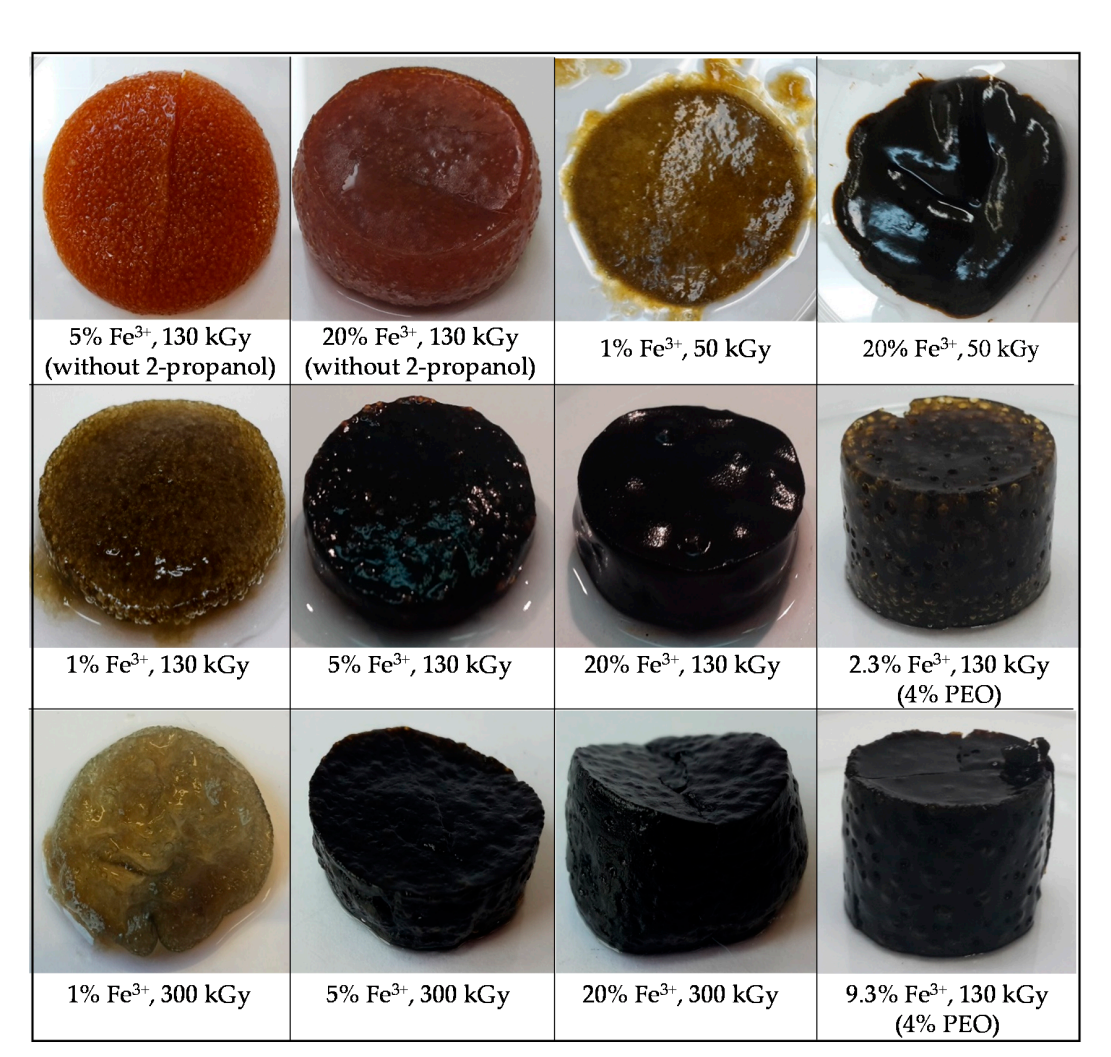
The photographs of PEO/Fe-oxide nanocomposite gels obtained from suspension with various Fe^3+^ content at various doses. Unless otherwise indicated, the precursor suspensions were prepared from 1.85 wt% PEO solutions and with the addition of 2-propanol (0.2 M).

Upon γ-irradiation, the reddish PEO/Fe(III) suspensions turned to reddish or white-green or black gels or black suspensions depending on the dose, 2-propanol concentration and the amount of Fe^3+^. The photographs of formed nanocomposite gels are shown in Figure 2.

**Figure 3:**
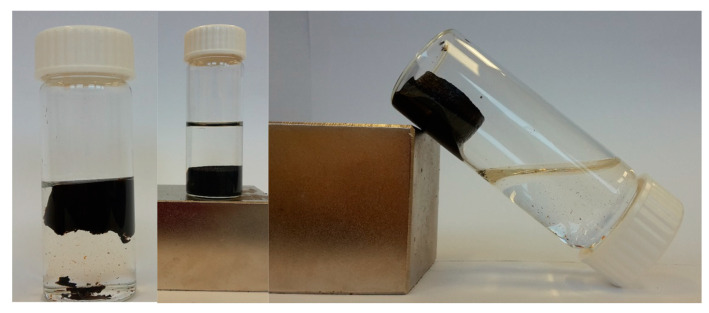
The photographs of magnetic PEO/iron oxide gel attracted by a permanent magnet.

γ-irradiation of pure PEO solutions (1.85 wt%) and PEO/Fe(III) precursor suspensions at pH ~ 12 without the addition of 2-propanol resulted in the formation of a permanent shape “wall-to-wall” macroscopic gels. The resulting nanocomposite gels were reddish-brown and non-magnetic. The reddish color and non-magnetic behavior indicated that even at the highest dose of 300 kGy no significant reduction of Fe(III) occurred (Figure 2). The reducing conditions were improved by the addition of 2-propanol (0.2 M). Irradiation of the pure 1.85 wt% PEO solution in the presence of 2-propanol did not lead to gel formation. On the other hand, the irradiation of PEO/Fe(III) precursor suspensions at pH~12 in the presence of 2-propanol led to the formation of nanocomposite gels (Figure 2). The white-green gels were obtained from 1 wt% Fe^3+^ suspensions, while the black magnetic hydrogels were obtained upon irradiation of 5 and 20 wt% Fe^3+^ suspensions at higher doses (130 and 300 kGy). The gel obtained from 20% Fe^3+^ suspensions at 300 kGy was strongly attracted by a permanent magnet as shown in Figure 3.

**Figure 4:**
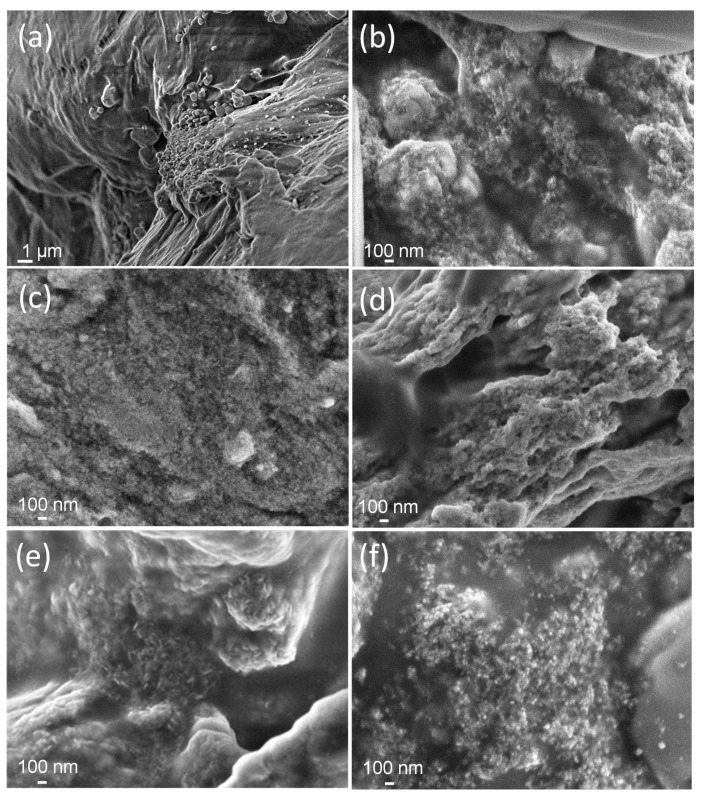
SEM micrographs of PEO/Fe-oxide gels obtained from suspensions with various Fe^3+^ content at various doses: (**a**) 5 wt% Fe^3+^ at 50 kGy; (**b**) 20 wt% Fe^3+^ at 50 kGy; (**c**) 5 wt% Fe^3+^ at 130 kGy; (**d**) 20 wt% Fe^3+^ at 130 kGy; (**e**) 5 wt% Fe^3+^ at 300 kGy; (**f**) 20 wt% Fe^3+^ at 300 kGy. All precursor suspensions were prepared from 1.85 wt% PEO solutions and with 0.2 M 2-propanol.

Figure 4 shows FE SEM images of composite gels obtained upon irradiation of suspensions with 2-propanol. The nanoparticles embedded into the polymer matrix are visible. Particles were mainly spherical in shape. The gel at 300 kGy and 20 wt% Fe^3+^ (Figure 4f) consisted of numerous spherical particles and/or particle aggregates of 40 nm in size homogeneously distributed throughout the polymer matrix. The SEM image of gel at 300 kGy and 5 wt% Fe^3+^ (Figure 4e) reveals the presence of larger irregular plate-like particles 70 nm in size, in addition to much smaller spherical ones.

**Figure 5:**
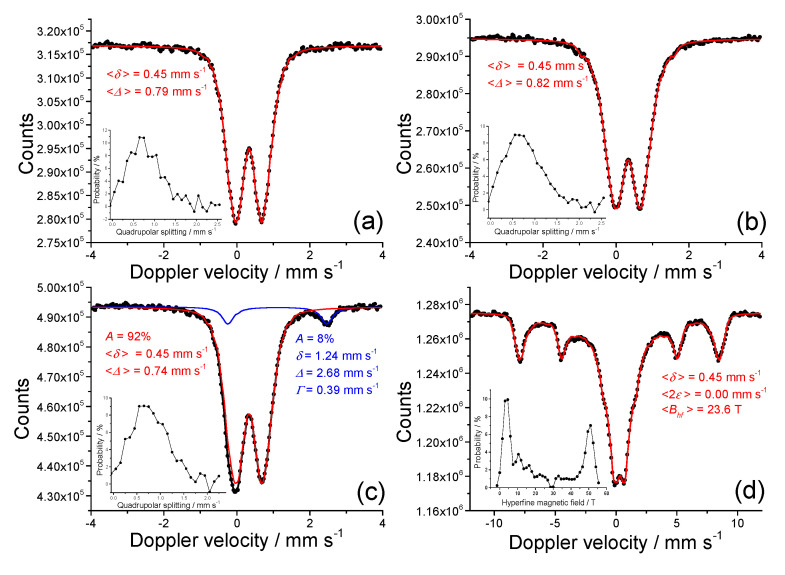
The 77 K Mössbauer spectra of PEO/Fe-oxide gels obtained from suspensions with: 5 wt% Fe^3+^ at 130 kGy (**a**); 20 wt% Fe^3+^ at 130 kGy (**b**); 5 wt% Fe^3+^ at 300 kGy (**c**); 20 wt% Fe^3+^ at 300 kGy (**d**). All precursor suspensions were prepared from 1.85 wt% PEO solutions and with 0.2 M 2-propanol. Mössbauer parameters are given: *δ* = isomer shift relative to α-Fe at 20 °C; *Δ* = quadrupole splitting; *Γ* = line width; *B*_hf_ = hyperfine field, A = relative area. *Δ* values are given as an average value of quadrupole splitting distributions (**a**–**c**). *B*_hf_ value is given as an average value of hyperfine field distribution (**d**). Line width values were fixed during the fitting of quadrupole splitting distribution (**a**–**c**) or hyperfine field distribution (**d**). Error: *δ* = ± 0.01 mm s^−1^; *Δ* = ± 0.01 mm s^−1^; *B*_hf_ = ± 0.3 T.

Figure 5 shows the Mössbauer spectra recorded at 77 K of nanocomposite gels obtained from suspensions with 2-propanol at 130 kGy and 300 kGy. Generally, the Mössbauer spectrometry is very sensitive to the presence of Fe(II) in inorganic [25,36] and biological samples [37]. The sample 5% Fe^3+^ at 130 kGy (Figure 5a) consisted of a symmetric doublet which was fitted with quadrupole splitting distribution. Even at this low-temperature the sample was not magnetically ordered, which reveals the ultrasmall particle size. This quadrupole splitting distribution can be ascribed to a poorly crystalline, disordered structure (average quadrupole splitting *<Δ*> for 5% Fe sample is 0.79 mm s^−1^ (Figure 5a) and for 20% Fe sample is 0.82 mm s^−1^ (Figure 5b)). Average isomer shift values of *<δ>* = 0.45 mm s^−1^ for both samples are consistent with those of Fe^3+^ [38]. The spectrum of gel obtained at 300 kGy and 5% Fe^3+^ consisted of a quadrupole splitting distribution component and the doublet whose parameters correspond to Fe^2+^ (*δ* = 1.24 mm s^-1^, *Δ* = 2.68 mm s^−1^) (Figure 5c). The gel obtained at 300 kGy and 20% Fe^3+^ exhibited a collapsing sextet (Figure 5d). Such spectrum is generally found with systems that exhibit superparamagnetic relaxation phenomena. This spectrum was best fitted with a distribution of the hyperfine magnetic field (average <*B*_hf_> was 23.6 T). The fit resulted in a bimodal distribution, which indicates that the sample consists of two types of particles; a population with the bigger particle size whose magnetic relaxation time is longer than the measurement time of ^57^Fe Mössbauer spectrometry (5 × 10^−8^ s), and a population of smaller particles whose relaxation time is somewhat shorter than the measurement time of Mössbauer spectrometry. The symmetric nature of the spectrum suggests that there is no Fe^2+^ in the sample. Furthermore, the fact that the isomer shift value is 0.45 mm s^−1^ and quadrupole shift is 0.00 mm s^−1^, which are the usual values for poorly crystalline maghemite sextet at 77 K [39], indicates that the sample is composed of maghemite. The spectra measured at 300 K are shown in Figure S1 in the Supplementary Materials (all spectra at room temperature are consistent with superparamagnetic and/or paramagnetic doublets).

**Figure 6:**
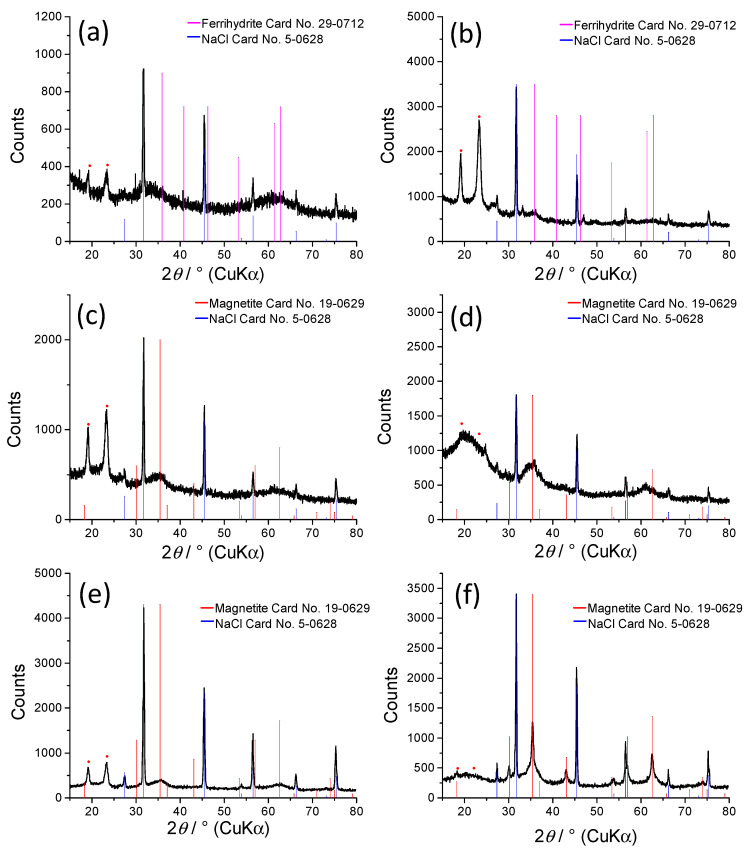
XRD patterns of unirradiated precursor (**a**) and PEO/Fe-oxide gels obtained from suspensions with: 5 wt% Fe^3+^ at 50 kGy (**b**), 5 wt% Fe^3+^ at 130 kGy (**c**), 5 wt% Fe^3+^ at 300 kGy (**d**) 20 wt% Fe^3+^ at 130 kGy (**e**) and 20 wt% Fe^3+^ at 300 kGy (**f**), at pH ~ 12. The two maxima at ~19 and ~23 degrees marked with red dots correspond to crystalline PEO.

Figure 6 presents the XRD patterns of unirradiated precursor (Figure 6a) and PEO/Fe-oxide gels obtained upon irradiation of suspensions with 2-propanol (0.2 M) and 5 wt% Fe^3+^ at 50 kGy (Figure 6b), 130 kGy (Figure 6c) and 300 kGy (Figure 6d), as well as 20 wt% Fe^3+^ at 130 kGy (Figure 6e) and 300 kGy (Figure 6f). The XRD patterns (Figure 6a–e) show two maxima which can be attributed to iron oxide phases. The XRD patterns of the precursor and the gel obtained at 50 kGy (Figure 6a,b) were ascribed to ferrihydrite and NaCl as an impurity. The XRD patterns of gels obtained at 130 and 300 kGy (Figure 6a–e) were attributed to magnetite NPs. On the other hand, the gel obtained from 20 wt% Fe^3+^ suspension at 300 kGy (Figure 6f) had sharper and more distinct maxima, indicating improved crystallinity of the formed magnetite NPs, which is in line with the results of Mössbauer spectrometry. The distinct maxima at ~19 and ~23° on the XRD patterns of composite gels obtained at 50 and 130 kGy are the result of partial crystallization of PEO gels upon drying. These maxima completely disappeared on the XRD pattern of nanocomposite gel obtained at 300 kGy and only wide amorphous halo is visible, due to the very dense crosslinking density of gel obtained at 300 kGy. The results of line broadening analyses are given in Table 1. The volume-averaged domain sizes (*D*_v_) of the dominant crystalline phase in the synthesized samples were estimated using the Scherrer equation:

*D*_*hkl*_ = 0.9*λ*/(*β_hkl_* × cos*θ*) (2)

where *D_hkl_* is the volume average domain size in the direction normal to the reflecting planes (*hkl*), *λ* is the X-ray wavelength (Cu*K*α), *θ* is the Bragg angle, and *β_hkl_* is the pure full width of the diffraction line (*hkl*) at half the maximum intensity. The volume-average domain size (*D*_v_) of the 110 lines (*D*_110_) of ferrihydrite in the unirradiated precursor was estimated to 1.7, whereas upon γ-irradiation to 50 kGy the average domain size increased to 4.5 nm (Table 1). At 130 kGy and 300 kGy (5 wt% Fe^3+^) ferrihydrite transformed to magnetite of about 2.3 nm in size (*D*_311_ ≅ 2.3 nm). The sample irradiated with the dose of 130 kGy with 20 wt% Fe^3+^ had a somewhat larger crystallite size (*D*_311_ ≅ 3.3 nm) than the sample prepared from suspension with lower Fe^3+^ concentration, which is expected. However, at 300 kGy and 20 wt% Fe^3+^ the crystallite size of magnetite nanoparticles increased significantly when compared to the 130 kGy sample. This significant increase in the crystallinity of nanoparticles was not observed for gels prepared from suspensions lower initial Fe^3+^ concentration where the magnetite crystallite size did not change noticeably.

**Figure 7:**
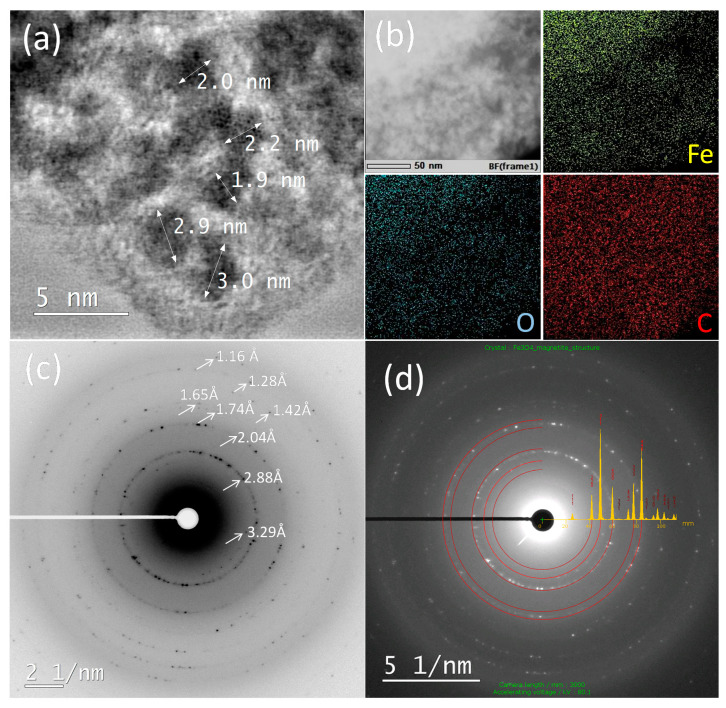
STEM bright-field image of the gel obtained at 130 kGy from 20 wt% Fe^3+^ suspension (**a**); EDXS elemental mapping of the gel where yellow, blue, and red colors represent iron (Fe), oxygen (O), and carbon (C), respectively (**b**); SAED (selected area electron diffraction) of spherical nanoparticles (NPs) with marked interplanar distances (**c**); SAED of spherical NPs indexed as magnetite (Fe_3_O_4_) (**d**).

TEM analysis of gel obtained at 130 kGy from suspension with 20 wt% Fe^3+^ is shown in Figure 7. Figure 7a shows the STEM bright-field micrograph of the gel with slightly high-frequency FFT filtered BF-STEM image of very thin area (where individual Fe atom columns can bee seen in some nanoparticles). The very small (~3 nm) particles can be seen confirming the results obtained by XRD line-broadening analysis. The much larger particle size observed using SEM in the scattering mode (Figure 4) compared to the TEM determination in the transmission mode (Figure 7a) is because the SEM sees the iron oxide nanoparticles “disguised” by polymer whereas the TEM can see the “pure” individual iron oxide nanoparticles. EDXS (energy-dispersive X-ray spectroscopy) elemental mapping (Figure 7b) shows that three major elements are iron, oxygen, and carbon, and that they are homogeneously distributed throughout the sample indicating good dispersion of nanoparticles within the gel matrix. SAED (selected area electron diffraction) patterns (Figure 7c,d) match magnetite and NaCl impurity thus confirming the results of XRD, i.e., the formation of magnetite nanoparticles inside the PEO gel. The Mössbauer results suggested that magnetite nanoparticles were completely oxidized to maghemite. This discrepancy arises because the SAED measures the sample in high vacuum under reducing conditions. On the contrary, Mössbauer spectrometry is very sensitive to Fe(II) at the ambient conditions, and as a rule the Mössbauer spectrometry for small nanoparticles below 5 nm shows no Fe(II). This is because very small magnetite nanoparticles can easily be oxidized in air.

**Figure 8:**
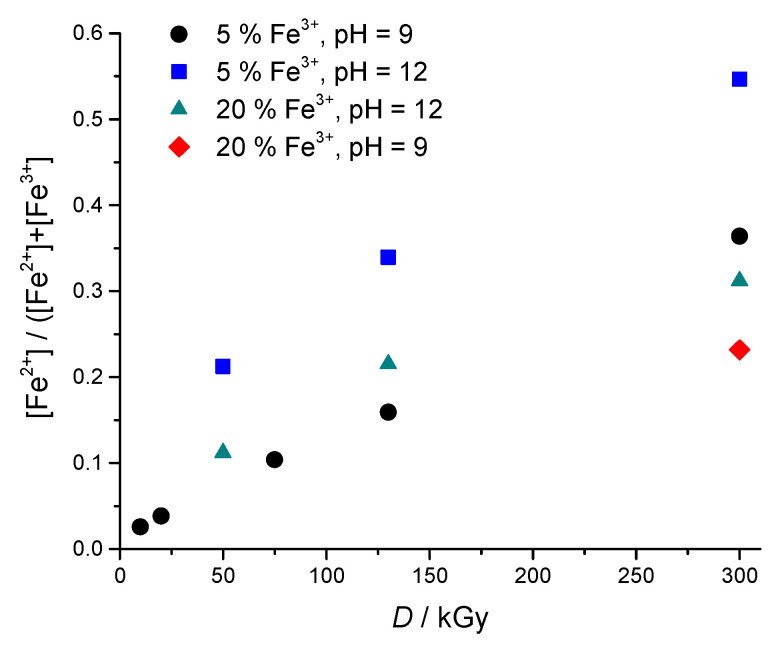
The Fe^2+^ fraction ([Fe^2+^/(Fe^2+^+Fe^3+^)]) in relation to dose, pH and initial amount of Fe^3+^ in precursor suspensions as determined using the 1,10-phenanthroline UV-Vis spectrophotometric method. Samples were obtained from 1.85 wt% PEO precursor suspensions with 0.2 M 2-propanol.

The amount of Fe^2+^ generated upon γ-irradiation was quantitatively determined using 1,10-phenanthroline UV-Vis spectrophotometric method. Figure 8 shows the Fe^2+^ fraction ([Fe^2+^/(Fe^2+^+Fe^3+^)]) in the γ-irradiated samples prior to their isolation and coming in contact with air. This was obtained by the addition of concentrated HCl immediately after irradiation through rubber septa (described in experimental). The Fe^2+^ molar fraction depended on dose, pH and initial concentration of Fe^3+^ in precursor suspensions. The Fe^2+^ fraction increased with the increase of dose and pH and with the lower initial Fe^3+^ concentration. At 130 kGy and initial 5 wt% Fe^3+^ the reduction yield was 34% in comparison to 22% in gels obtained from 20 wt% Fe^3+^ suspension. The reduction yield of 31% obtained for the sample at 300 kGy from 20 wt% Fe^3+^ suspension resulted in a highly magnetic gel. The highest reduction yield of Fe^2+^ (54.7%) was achieved at the dose of 300 kGy, pH = 12 for 5% Fe^3+^ precursor suspension. It should be noted that at similar experimental conditions the γ-irradiation in the presence of dextran sulfate or DEAE-dextran polymer generated almost 100% of Fe^2+^ at 130 kGy [25,35]. More reducing conditions were obtained at higher pH. This can be explained by the higher yield of hydrated electrons in a highly alkaline medium [19,40,41,42,43]. In an alkaline medium, the hydrogen atoms are converted to hydrated electrons. Because of the fact that the higher reducing conditions were obtained at higher pH, all investigated gels were synthesized at pH ~ 12.

**Figure 9:**
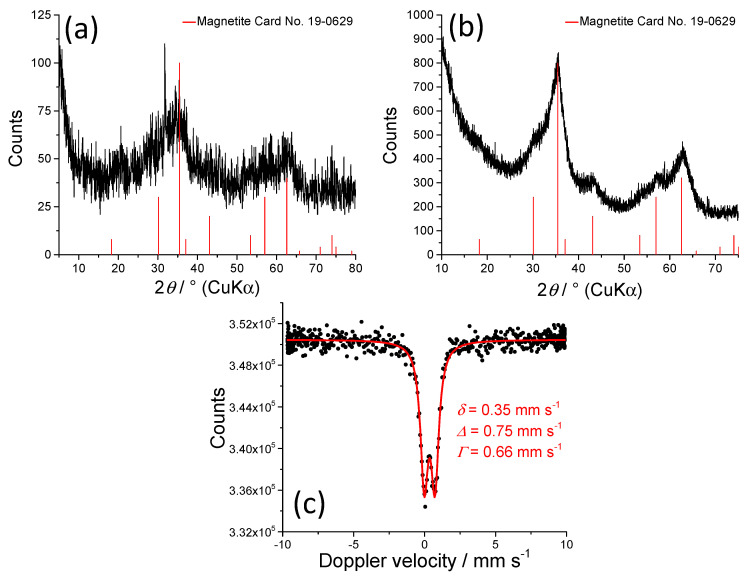
XRD patterns and Mössbauer spectrum at room temperature of PEO/Fe-oxide black magnetic powders obtained from 1.85 wt% PEO suspensions with 20 wt% Fe^3+^ and four times more 2-propanol (0.8 M) at 50 kGy (**a**) and 130 kGy (**b**,**c**).

The increase of 2-propanol concentration in initial suspensions to 0.8 M did not result in the formation of gels, but in the formation of black magnetic suspensions. Figure 9 shows the XRD patterns and Mössbauer spectrum at room temperature of black magnetic powders isolated from suspensions obtained upon irradiation of 20 wt% Fe^3+^ suspensions with 0.8 M 2-propanol at 50 kGy (Figure 9a) and at 130 kGy (Figure 9b,c). These samples consist of magnetite with the volume-average domain size of approximately 3 nm in size (Table 1). The doublet in the Mössbauer spectrum, which can be assigned to small superparamagnetic maghemite (oxidized magnetite nanoparticles), is in accordance with XRD line broadening analysis (*D*_311_ = 3.2 nm).

As can be seen from the above results, a critical step in the one-step synthesis of magnetic iron oxide nanocomposite gels was to find a balance between the good reducing conditions required for the formation of magnetic particles and the conditions suitable for the formation of the polymer network. The permanent shape of wall-to-wall gels obtained on irradiation without 2-propanol is strong evidence of a three-dimensional network and PEO intermolecular crosslinking. It is known that on irradiation of dilute PEO aqueous solutions, the main mechanism for crosslinking of PEO chains is a reaction with hydroxyl radicals formed on water radiolysis (Equation (1)) [31,32,44]. If conditions are favorable (a relatively low dose rate and a diluted PEO concentration), owing to good mobility in dilute solutions, such formed PEO macroradicals preferably crosslink resulting in macrogel formation.

CH_2_-CH_2_-O-CH_2_-CH_2_-O- + ^•^OH (H^•^) → -CH_2_-CH_2_-O-CH_2_-CH^•^-O- + H_2_O (H_2_) (3)

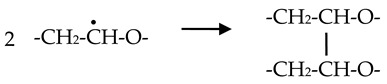
(4)

The precursor particles did not significantly disturb the intermolecular crosslinking of PEO chains. Although during the process of formation of PEO network, ^•^OH radicals, which are oxidizing agents, are partially removed from the system, the reducing conditions when irradiated without 2-propanol were still not strong enough to reduce the Fe(III) precursor nanoparticles. When 2-propanol in a concentration of 0.2 M was added in a system to enhance reducing conditions, magnetite nanoparticles were formed (Figure 5, Figure 6, Figure 7 and Figure 8). 2-propanol scavenges hydroxyl radicals which are oxidizing agents, and prevents back oxidation of formed ferrous ions

^•^OH (H^•^) + (CH_3_)_2_CHOH → H_2_O (H_2_) + (CH_3_)_2_C^•^OH (5)

Thus formed 2-propanol radicals can act as additional reducing agent [45]:

Fe^3+^ + (CH_3_)_2_C^•^OH → Fe^2+^ + (CH_3_)_2_CO + H^+^ (6)

The addition of 2-propanol had a pronounced effect on the formation of PEO gels. Irradiation of pure PEO solutions with 2-propanol (0.2 M) did not result in the formation of any gel content, even at 300 kGy. By scavenging the ^•^OH radicals, which are also initiators of PEO crosslinking, 2-propanol reduced the yield of PEO macroradicals, and hence the crosslinking degree. On the other hand, on irradiation of PEO/Fe(III) precursor suspensions, magnetic gels were formed, and the amount of gel depended on the dose and initial amount of Fe^3+^ (Figure 2). This suggests that PEO chains are additionally crosslinked through formed iron oxide nanoparticles. An additional reason may lie in the possible contribution of iron oxide NPs to the formation of higher yield of hydroxyl radicals, like the one observed for nanosilica by Le Caër et al. [46]. They showed that on irradiation of the silica/water system, an exciton was formed that may be scavenged by water molecules and further react to produce additional hydroxyl radicals, protons, and hydrogen. Besides, the additional hydroxyl radicals may be formed in the Fenton reaction, because the Fe^3+^ that is reduced to Fe^2+^ by hydrated electrons may be reoxidized by radiolytically formed H_2_O_2_ thus producing additional ^•^OH radicals.

At higher 2-propanol concentrations (0.8 M) there were not enough hydroxyl radicals left for the polymer to crosslink, resulting in the formation of highly magnetic magnetite NPs suspensions (Figure 8).

When more concentrated PEO suspensions (4 wt%) pure or with various amount of Fe^3+^ were irradiated, black wall-to-wall hydrogels were obtained, similar to those obtained by irradiation without 2-propanol, probably due to the higher yield of PEO intermolecular crosslinking (photos in Figure 2).

Therefore, by finding the optimal conditions we were able to synthesize magnetic PEO/iron oxide nanocomposite gels in a single step.

### 3.2. Thermal Characterization of Gels

**Figure 10:**
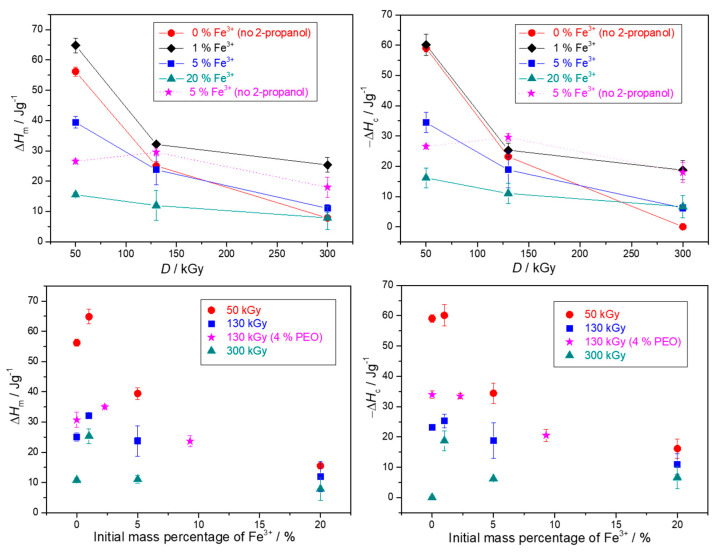
Melting (Δ*H*_m_) and crystallization (Δ*H*_c_) enthalpies of the 2nd heating cycles and 1st cooling cycles, respectively, of the obtained gels in dependence on the irradiation dose and the mass percentage of Fe^3+^ in precursor suspensions. Unless otherwise indicated, the precursor suspensions were prepared from 1.85 wt% PEO solutions and with 0.2 M 2-propanol. Mass percentage of Fe^3+^ of 1, 5, 20 wt% (relative to total PEO and Fe^3+^ mass) in the precursor suspensions correspond to concentrations of Fe^3+^ ions of 0.35 × 10^−2^ M, 1.75 × 10^−2^ M, and 7 × 10^−2^ M in the case of 1.85 wt% PEO suspensions. In the case of 4 wt% PEO suspensions, Fe^3+^ concentrations of 1.75 × 10^−2^ M and 7 × 10^−2^ M correspond to 2.3 and 9.3 wt% Fe^3+^.

**Figure 11:**
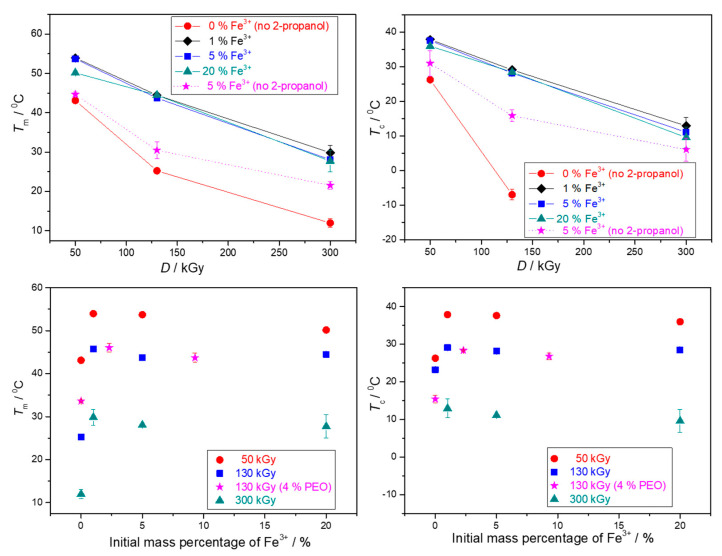
Melting (*T*_m_) and crystallization (*T*_c_) temperatures of the 2nd heating cycles and 1st cooling cycles, respectively, of the obtained gels in dependence on the irradiation dose and the mass percentage of Fe^3+^ in precursor suspensions. Unless otherwise indicated, the precursor suspensions were prepared from 1.85 wt% PEO solutions and with 0.2 M 2-propanol.

The thermal properties of obtained nanocomposite gels in dependence on the γ-irradiation dose and the initial Fe^3+^ concentration were studied by differential scanning calorimetry. The DSC thermograms are given in Figure S2 in Supplementary Materials, while the phase transformation enthalpies and temperatures of the second heating and first cooling cycles are given in Figure 10, Figure 11, Figures S3 and S4. The results are presented in dependence on both the irradiation dose and the mass percentage of Fe^3+^ in precursor suspensions, as some effects are easier to observe. It must be emphasized again that when comparing the results of pure PEO gels with those of nanocomposites, one has to take into account that pure PEO gels were formed on irradiation without 2-propanol, because with 2-propanol in pure PEO solutions gels were not formed. Therefore, the difference between pure PEO gels and nanocomposite gels is not solely due to the amount of NPs within the gel, but also due to the influence of 2-propanol on network formation.

Enthalpies and temperatures of melting and crystallization decreased with the dose over the entire dose range for both pure PEO gels and PEO/iron oxide nanocomposite gels (Figures 10, Figure 11, Figures S3 and S4). At 300 kGy almost completely amorphous gels with a high degree of crosslinking were obtained, especially in the case of pure PEO gel and gel obtained from 20 wt% Fe^3+^ suspension (Figure 10). At higher doses there is a higher yield of PEO intermolecular crosslinking; crosslinks are dense enough to significantly restrain mobility and impede crystallization of PEO chains on drying, which resulted in more amorphous gels. The smallest changes of enthalpies in dependence on dose were for gels with the highest NPs content (highest initial Fe^3+^ content) (Figure 10). The decrease of melting (*T*_m_) and crystallization (*T*_c_) temperatures with the dose was the most abrupt for pure PEO gels (Figure 11). A high density of crosslinks is the main reason for such low *T*_m_ and *T*_c_. Crosslinks increase the number of defects in the crystalline phase resulting in less “perfect“ crystallites and consequently decrement in the melting temperature. Crosslinks also impose restrictions on molecular motions of PEO chains and at high doses the high density of PEO crosslinks and a small segment of PEO chains between two crosslink junctions seriously impede crystallizability, resulting in a significant lowering of *T*_c_. Such a decrease of *T*_m_ and enthalpies of melting with the dose for PEO gels was also reported by other authors [12,30,47].

The increased amount of initial Fe^3+^ salt at the same dose led to the increased amount of formed gel with reduced enthalpies, suggesting the enhanced PEO crosslinking through formed Fe-oxide NPs (Figure 10 and Figure S4). In general, all nanocomposite gels at a certain dose had higher melting and crystallization temperatures than pure PEO gels (Figure 11). The amount of nanoparticles (initial Fe^3+^ content) at a certain dose had a significant effect on enthalpies decrease, but very little impact on temperatures (Figure 10). While there was approximately a 20 °C jump in *T*_m_ and *T*_c_ for gel obtained from 1% Fe^3+^ suspension compared to pure PEO gels at the certain dose, the further increase of initial Fe^3+^ to 20% resulted in a higher amount of formed gels with lower enthalpies but only slightly lower *T*_c_ and *T*_m_. A similar trend in temperature changes with the increase of silica NPs we observed in our previous work [12].

The observed change in the melting enthalpies and temperatures with the NPs content can be explained by the impact of NPs. NPs can act as nucleating agents—they induce heterogeneous crystallization centers and thus facilitate crystallization of uncrosslinked PEO segments and increase *T*_c_ [48]. On the other hand, at high NPs concentration, due to worsened dispersion and partial NPs agglomeration, NPs can restrain the mobility of partially crosslinked PEO chains and its crystallizability. At the same time, agglomerates can be a serious obstacle for PEO crosslinking. Since in composites obtained with 2-propanol, more gel was formed with initial Fe^3+^ increase, the main reason for the decrease in enthalpies is obviously the additional crosslinking of PEO chains by NPs. So, while pure PEO gels are formed by intermolecular crosslinking of PEO chains, in nanocomposites some type of interactions/bonding between Fe-oxide NPs and PEO chains such as H-bonds or coordination bonding [49,50,51] are likely to contribute to the radiation-induced crosslinking of PEO chains, resulting in further amorphization. The highest dose dependence of enthalpies and temperatures for pure PEO gels support the above conclusion. The interactions of Fe_2_O_3_ and TiO_2_ particles and polymer have been observed observed by Popescu et al. [52], whereas Davenas et al. [53] noted that silica filler can act as an additional crosslinker that upon irradiation formed covalent bonds to the polymer matrix. Similarly, Agrawal et al. [54] observed laponite nanoparticles acting as additional junction points in physically associative PLA-PEO-PLA gel. Criado-Gonzalez et al. [55] and Peng at al. [56] observed the formation of a weak network due to crosslinking through coordination bonding of Fe(III) cations with alginate chains and PAA chains, respectively.

The role of NPs as nucleating agents can also be inferred from the behavior of gels obtained without 2-propanol. For nanocomposite gels prepared without 2-propanol the sole effect of nanoparticle content can be observed compared to the pure PEO gels (Figure 10, Figure 11and Figure S4).

The pure PEO gels and nanocomposite gels prepared from 4 wt% PEO suspensions had higher melting and crystallization enthalpies and temperatures than those obtained from 1.85 wt% PEO solutions at the same dose (Figure 10 and Figure S3). For pure PEO gels exactly the opposite would be expected; that irradiation of a more concentrated polymer solution would result in a higher crosslinks density and a more amorphous gel. The phase transformation temperatures of nanocomposite gels containing the same NPs content did not depend on PEO concentration, they were almost the same for 1.85 and 4 wt% suspensions.

### 3.3. Rheological Properties of Gels

The rheological properties of the gels were investigated at different conditions within the linear viscoelastic region (LVR).

**Figure 12:**
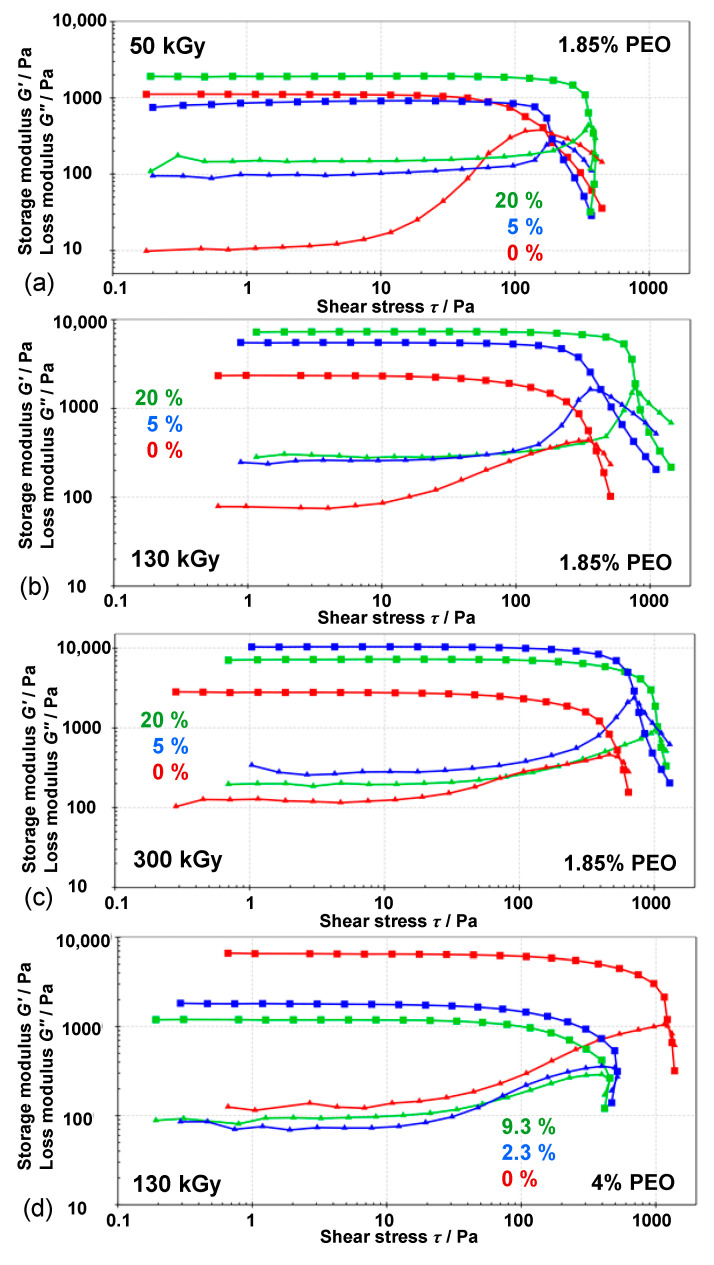
Amplitude sweep test (*G*′ (■) and *G*″ (▲) values) of pure PEO gels and nanocomposite gels obtained at (**a**) 50 kGy, (**b**) 130 kGy, and (**c**) 300 kGy from 1.85 wt% PEO suspensions, and at (**d**) 130 kGy from 4.0 wt% PEO suspensions, at 25 °C. The initial mass percentage of Fe^3+^ (relative to the total PEO and Fe^3+^ mass) in precursor suspensions is indicated.

The amplitude sweep test at room temperature for gels obtained at various doses starting from 1.85 wt% PEO precursor suspensions is presented in Figure 12a,b,c and Figure S5 in Supplementary Materials and the values are given in Table 2. All investigated gels behave like viscoelastic solids with *G*′ values (storage modulus) higher than *G*″ values (loss modulus), confirming a well-ordered gel network [57]. Storage moduli (as well as yield and flow point) increased with the irradiation dose for both pure PEO gels and all nanocomposite gels indicating higher network density. Slightly stiffer gels were formed at higher dose. The increase of the storage modulus *G*′ and crosslink density with the irradiation dose has been observed for different radiation crosslinked gels, like PEO [58] and PVP hydrogels [59]. All nanocomposite gels had higher storage moduli, yield points and flow points compared to pure PEO gels at a certain dose. Generally, at a particular dose, storage moduli, yield point and flow point increased with the increase of Fe^3+^ content in precursor suspensions. At 50 and 130 kGy storage moduli increased with initial Fe^3+^ content, while at 300 kGy there was an extreme increase for gel prepared from 5 wt% Fe^3+^ suspension compared to pure PEO gel but no further increase for gel prepared from 20 wt% Fe^3+^ suspension. The quantity loss factor, tan(*δ*) = *G*″/*G*′, determines the relative elasticity of viscoelastic materials. The gels with a value of tan(*δ*) = 0.1 and lower belong to stiff gels and are indicative of well-ordered systems. For 50 kGy loss factor value was low for pure PEO (0.01) compared to gels prepared from 5 % (0.11) and 20% (0.08) Fe^3+^ suspensions, meaning that pure PEO has a better ordered microstructure of the gel compared to nanocomposites. At 130 kGy the values of storage modulus, the yield, and flow points of gel obtained at 20% initial Fe^3+^ content were 2 to 4 times higher than for gel from initial 5 wt% Fe^3+^ and pure PEO, respectively, but the loss factor values of these gels were similar. The gel obtained from suspensions with the highest Fe^3+^ content (20%) at 300 kGy showed the longest yielding zone (the zone between yield point and flow point) through a range of 700 Pa indicating very stiff structure.

The results show that a higher degree of intermolecular crosslinking with increased dose increases the storage modulus and the gel becomes stiffer. But pure PEO with a high degree of exclusively intermolecular PEO crosslinking was still softer than nanocomposite gels. The increase in elastic moduli, flow, and yield points of nanocomposite gels and the formation of stronger gels confirms a well-ordered gel network, well-ordered microstructure and indicates good NPs dispersion. This increase is not only due to the reinforcing effect of inorganic NPs, but the formed magnetic NPs facilitate the formation of gels by acting as additional crosslinkers. Similarly, Agrawal et al. [54] found that *G*′ of PLA-PEO-PLA gel increased dramatically when the amount of laponite particles was increased, indicating the formation of new junctions by the nanoparticles. Blyakhman et al. [1] reported the increase in Young’s modulus of PAAm gel resulting from the addition of a low concentration of magnetic NPs, reflecting the direct effect of magnetic NPs on gel elasticity, and reported the possibility that MNPs act as crosslinking agents.

All this is consistent with the decrease in enthalpies and temperatures of these gels with increasing dose and Fe^3+^ content, as observed from DSC measurements. In addition, the similar values of *G*′ of 20% compared to 5% gels at 300 kGy (in line with the similar melting enthalpies) (Figure 12 and Figure S5) may be due to the likely agglomeration of NPs at high NPs content.

The amplitude sweep test of gels obtained at 130 kGy from 4 wt% PEO precursor suspensions showed the opposite behavior depending on the initial Fe^3+^ content (Figure 12d) to gels obtained from 1.85 wt% PEO suspensions. The gels obtained from 4 wt% PEO suspensions had the same initial concentration of Fe^3+^ salt as in 1.85 wt% PEO (resulting in 2.3 and 9.3 wt% Fe^3+^ relative to the total initial mass of PEO and Fe^3+^). The pure PEO gel obtained from 4 wt% PEO was by far the strongest by all parameters, while the gel with the highest NPs content was the weakest. *G*′ values, yield point and flow point were in descending order with increasing initial Fe^3+^ amount, while the loss factor (tan *δ*) increased. As expected, stronger gels can be obtained by increasing the polymer concentration in the starting suspension, resulting in a higher density of the network formed by intermolecular crosslinking. But the effect of NPs did not show an improved behavior as in the case of lower polymer concentration. Obviously, at higher polymer concentration the effect of intermolecular crosslinking of PEO chains is dominant over crosslinking through Fe-oxide NPs.

**Figure 13:**
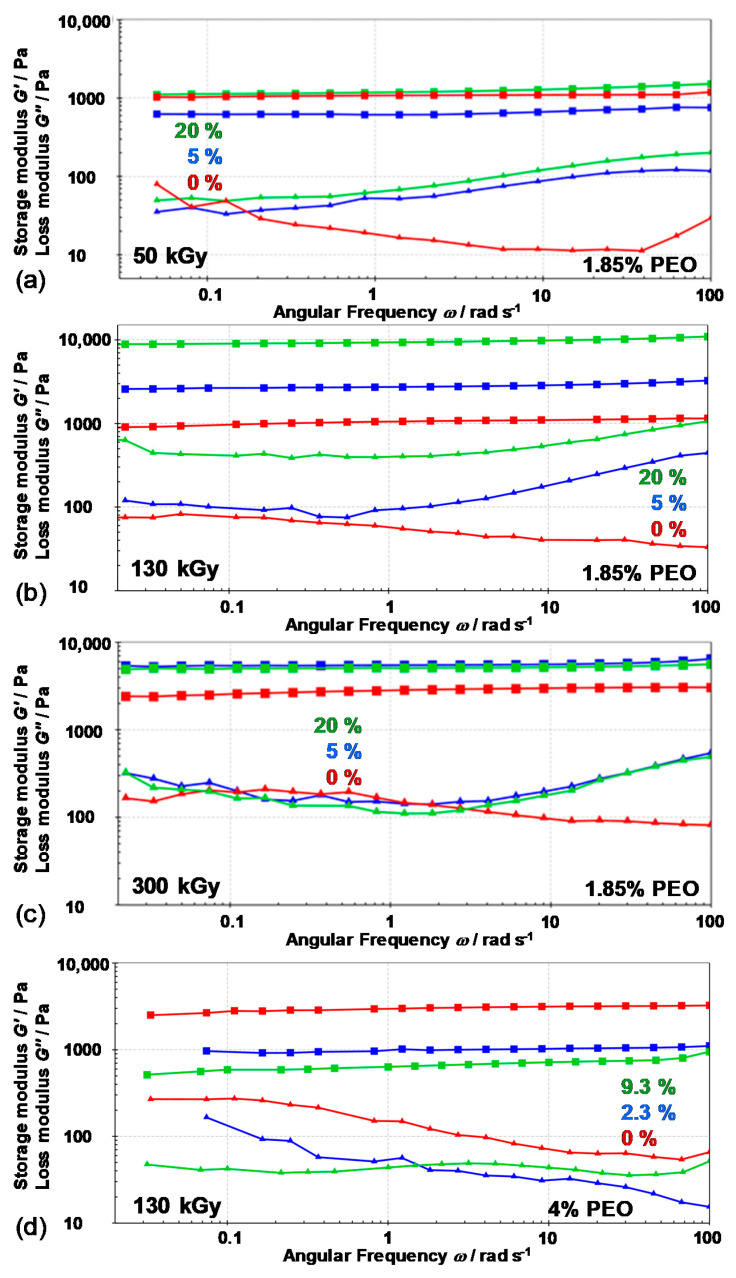
Frequency sweep tests (*G*′ (■) and *G*″ (▲) values) of pure PEO gels and nanocomposite gels obtained at (**a**) 50 kGy, (**b**) 130 kGy, and (**c**) 300 kGy from 1.85 wt% PEO suspensions, and at (**d**) 130 kGy from 4.0 wt% PEO suspensions, at 25 °C. Initial mass percentage of Fe^3+^ in precursor suspensions is indicated.

Figure 13 presents the frequency sweep measurements. Frequency sweeps describe the time-dependent behavior of a sample in the non-destructive deformation range. The investigated samples are true chemically crosslinked gels. The frequency sweep of pure PEO and magnetic nanocomposite gels showed constant storage modulus (*G*′) values within the entire frequency range (100 rad/s to 0.05 rad/s). Pure PEO and PEO/Fe-oxide gels showed similar behavior at lower frequencies, but at higher frequencies, pure PEO gel obtained more ordered structure, better homogeneity (lower loss factor) compared to nanocomposite gels with higher loss factor values (because of increase of loss modulus at higher frequences). The same remarks related to internal gel microstructure have been seen for pure PEO gels and nanocomposites at different doses except for gels from 4 wt% of PEO.

**Figure 14:**
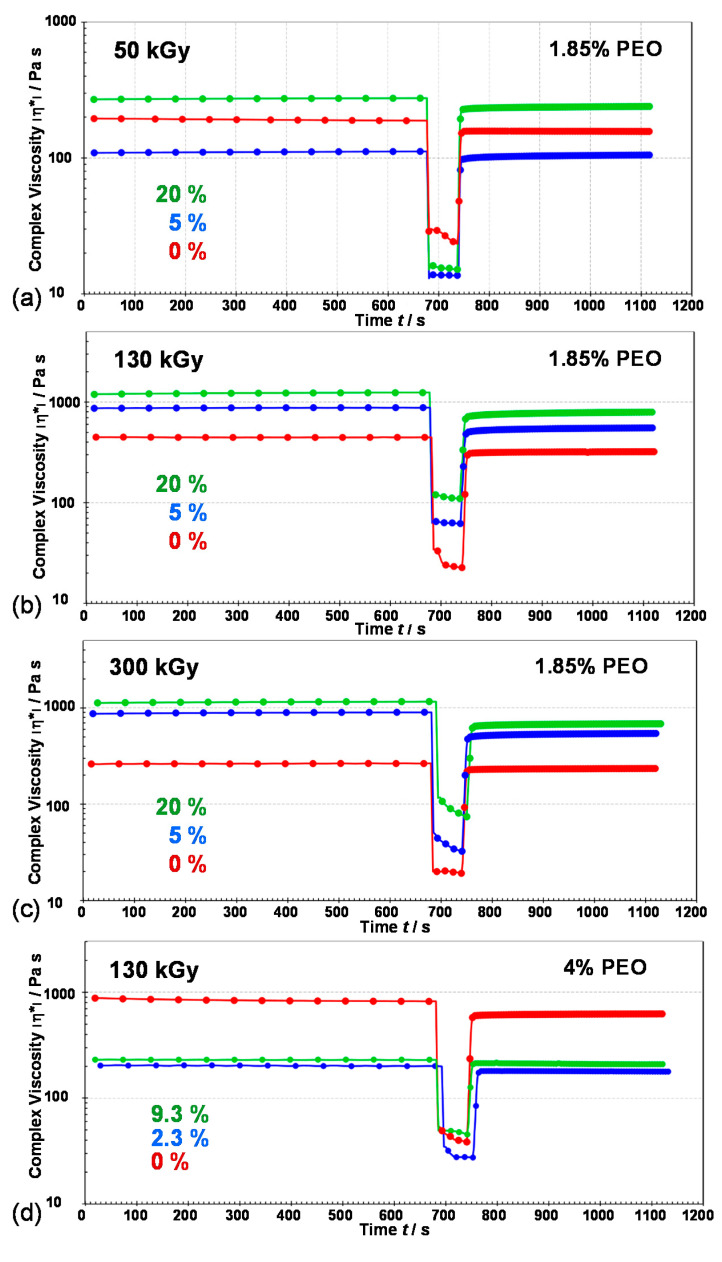
3-interval thixotropy test (3ITT) of pure PEO gel and nanocomposite gels obtained at (**a**) 50 kGy, (**b**) 130 kGy, (**c**) 300 kGy from 1.85 wt% PEO suspensions and (**d**) from 4 wt% PEO suspensions at 130 kGy showed complex viscosity (ղ*) as a function of time and application of different strains (LVR-DR-LVR) at 25 °C. Linear viscoelastic region (LVR): strain = 0.1%, frequency = 5 Hz; destructive region (DR): strain =300%, frequency = 5 Hz. Initial mass percentage of Fe^3+^ in precursor suspensions is indicated.

Results of 3ITT thixotropy test are given in Figure 14 and Figure S6. Thixotropy is a time-dependent phenomenon. It can be defined as the shear thinning behavior of a viscoelastic gel sample upon the application of a destructive strain and the subsequent recovery of the viscoelastic properties after cessation of the strain. Figure 14 shows the evaluation of complex viscosity and Figure S6 the evolution of *G*′ and *G*″ of gels after applying the destructive strain. The sample behavior switched from gel-like to sol-like, with *G*″ values higher than *G*′. After that, the original conditions reapplied and the recovery of viscoelastic properties of gels was observed. Recovery ratios of gels within 60 s are given in Table 3. The initial Fe^3+^ amount, that is the amount of NPs formed, and the density of the PEO network formed at a specific γ-irradiation dose influenced the self-recoverable properties of the new nanocomposite materials. The most prominent recovery of the viscoelastic properties was observed for gels from 1.85 wt% PEO suspensions at 50 kGy and gels from 4 wt% PEO suspensions at 130 kGy (Table 3). The recovery of pure PEO gel at the specific irradiation dose was always better compared to the nanocomposite gels (indicating very good microstructure integrity of pure PEO gel), except for gels obtained from 4 wt% PEO suspensions. The lowest recovery was observed for gel obtained at 300 kGy from 20% Fe^3+^ suspension (57.1% recovery in 60 s). Better recovery was observed for all gel samples with lower *G*′ values compared to the recoverable properties of the most potent gels.

The obtained magnetic PEO/iron oxide nanocomposite gels showed promising rheological properties for potential application in tissue engineering and as wound dressings. By further optimization of the system and irradiation conditions, magnetic gels with tailored properties for a particular application could be synthesized.

## 4. Conclusions

γ-irradiation proved to be a suitable method for the one-step synthesis of magnetic PEO/iron oxide nanocomposite hydrogels. For the one-step irradiation synthesis of magnetic PEO/iron oxide hydrogels the appropriate balance between conditions suitable for polymer crosslinking and conditions suitable for the reduction of Fe(III)-precursor was crucial.

γ-irradiation generated Fe^2+^ was quantitatively determined using the 1,10-phenanthroline UV-Vis spectrophotometric method. A maximum Fe^2+^ mole fraction of 55% was achieved at a dose of 300 kGy and with 5 wt% of the initially added Fe^3+^ at pH ~ 12.

The thermal, viscoelastic, and magnetic properties of the gels depended on the irradiation dose, and the PEO and initial Fe^3+^ concentration, i.e., amount of magnetic iron oxide NPs inside the gels. Stronger gels were formed at the higher dose and higher magnetite NPs content (in the case of 1.85 wt% PEO).

Both rheological measurements and DSC results suggested that the pronounced increase in strength and stiffness of nanocomposite gels was not only due to the reinforcing effect caused by the presence of iron oxide NPs, but that the formed magnetic iron oxide nanoparticles acted as additional crosslinkers of the PEO chains, thus facilitating the formation of potent gels.

At higher PEO concentrations (4 wt%), the effect of intermolecular crosslinking of PEO chains was dominant over the effect of Fe-oxide NPs.

γ-irradiation of aqueous suspensions containing PEO, Fe^3+,^ and 2-propanol in alkali offered a possibility to obtain magnetic PEO/iron oxide nanocomposite gels with low crystallinity and improved strength.

By further optimization of the system and irradiation conditions (pH, polymer molecular mass, polymer and precursor concentration, as well as dose and dose rate) magnetic gels with tailored properties for a specific application could be synthesized.

## Figures and Tables

**Table 1:** The volume-averaged domain size (*D*_v_) of the dominant crystalline phase of selected samples.

Sample	Phase	*hkl*	2*θ*/°	FWHM/°	*D_hkl_*/nm
unirradiated precursor (5 wt% Fe^3+^, 0.2 M 2-propanol)	ferrihydrite	110	~35	5.1	1.7
	PEO + halite (impurity)				
gel - 50 kGy (5 wt% Fe^3+^, 0.2 M 2-propanol)	ferrihydrite	110	35.8	1.9	4.5
	PEO + halite (impurity)				
gel - 130 kGy (5 wt% Fe^3+^, 0.2 M 2-propanol)	magnetite	311	35.5	3.6	2.3
	PEO + halite (impurity)				
gel - 300 kGy (5 wt% Fe^3+^, 0.2 M 2-propanol)	magnetite	-	35.3	3.5	2.4
	halite + unidentified impurity				
gel - 130 kGy (20 wt% Fe^3+^, 0.2 M 2-propanol)	magnetite	311	35.5	2.6	3.3
	PEO + halite (impurity)				
gel - 300 kGy (20 wt% Fe^3+^, 0.2 M 2-propanol)	magnetite	311	35.4	0.6	13.9
	PEO + halite (impurity)				
powder - 50 kGy (20 wt% Fe^3+^, 0.8 M 2-propanol)	magnetite	311	35.5	3.6	2.3
powder - 130 kGy (20 wt% Fe^3+^, 0.8 M 2-propanol)	magnetite	311	35.5	2.6	3.3

**Table 2:** Results of amplitude sweep test of pure PEO gels and PEO/Fe-oxide nanocomposite gels.

Initial Mass Percentage of Fe^3+^ */%	Mass Percentage of PEO Aqueous Solution/%	*D*/kGy	*G*′ (Max)/Pa	Yield Point/Pa	Flow Point/Pa	Loss Factor (tan *δ* = *G*″/*G*′)
0	1.85	50	816	56.0	166.3	0.01
5	1.85	50	1115	117.4	192	0.11
20	1.85	50	1907	192.8	375	0.08
0	1.85	130	2336	98.5	377.3	0.04
5	1.85	130	5519	201.9	439.2	0.05
20	1.85	130	7300	455.3	788.3	0.04
0	1.85	300	2784	124.2	559.7	0.04
5	1.85	300	10,325	386.3	736.3	0.03
20	1.85	300	7220	355.0	1086	0.03
0	4	130	6507	298.6	1261	0.03
2.3 **	4	130	1800	82.4	517.4	0.05
9.3 **	4	130	1200	72.8	450.2	0.09

Pure PEO gels were prepared by irradiation without the addition of 2-propanol. * Initial wt% of Fe^3+^ relative to the total mass of PEO and Fe^3+^ in precursor suspensions. ** 4 wt% PEO suspensions had the same initial concentration of Fe^3+^ salt as in 1.85 wt% PEO, i.e., suspensions contained ~2x more PEO but the same amount of Fe^3+^, resulting in 2.3 and 9.3 wt% of Fe^3+^ relative to the total PEO and Fe^3+^ mass.

**Table 3:** Self-recoverable properties of pure PEO gels and nanocomposite gels obtained at various doses and compositions of precursor suspensions determined in 3-interval thixotropy test.

wt% Fe^3+^	0	5	20	0	5	20	0	5	20	0	2.3	9.3
**wt% PEO**	1.85	1.85	1.85	1.85	1.85	1.85	1.85	1.85	1.85	4.0	4.0	4.0
**Dose (kGy)**	50	50	50	130	130	130	300	300	300	130	130	130
**Recovery** **3ITT test (%)** ***t* = 60 s**	96.1	90.7	84.7	70.8	59.7	60.5	87.0	57.6	57.1	74.6	90.4	92.6

## Supplementary Materials

The following are available online at https://www.mdpi.com/2079-4991/10/9/1823/s1, Figure S1: The room temperature Mössbauer spectra of PEO/Fe-oxide gels obtained from suspensions with: (a) 5 wt% Fe^3+^ at 130 kGy; (b) 20 wt% Fe^3+^ at 130 kGy; (c) 5 wt% Fe^3+^ at 300 kGy; (d) 20 wt% Fe^3+^ at 300 kGy. All precursor suspensions were prepared from 1.85 wt% PEO solutions and with 0.2 M 2-propanol. Mössbauer parameters are given: *δ* = isomer shift relative to α-Fe at 20 °C; *Δ* = quadrupole splitting; *Γ* = line width. Error: *δ* = ± 0.01 mm s^-1^; *Δ* = ± 0.01 mm s^-1^, Figure S2: DSC thermographs of the 2nd heating (a,b) and the 1st cooling (c,d) cycles of pure PEO gel and nanocomposite gels obtained at 50, 130 and 300 kGy from 1.85 wt% PEO precursor suspensions with various Fe^3+^ content. Unless otherwise indicated, suspensions contained 0.2 M 2-propanol, Figure S3: Melting enthalpies and temperatures of the 1st heating cycles of the obtained gels in dependence on the irradiation dose and the mass percentage of Fe^3+^ in precursor suspensions. Unless otherwise indicated, the precursor suspensions were prepared from 1.85 wt% PEO solutions and with addition of 0.2 M 2-propanol. All gels obtained at 300 kGy with 2-propanol, and pure PEO gel at 130 kGy, were totally amorphous in the first heating cycle (no melting enthalpies). Gels obtained by irradiation from 5 wt% Fe^3+^ suspensions without 2-propanol had two melting maxima (both are given on graph), Figure S4: Melting (Δ*H*_m_) and crystallization (Δ*H*_m_) enthalpies and temperatures (*T*_m_ and *T*_c_) of the 2nd heating cycles and the 1st cooling cycles, respectively, of gels obtained at various doses in dependence on the mass percentage of Fe^3+^ in 1.85 wt% PEO precursor suspensions. Unless otherwise indicated suspensions contained 0.2 M 2-propanol, Figure S5: Comparison of amplitude sweep test (*G*′ (■) and *G*″ (▲) values) of nanocomposite gels obtained at 130 kGy and 300 kGy (1.85 wt% PEO solution), at 25 °C. Initial mass percentage of Fe^3+^ in precursor suspensions is indicated, Figure S6: 3-interval thixotropy test (3ITT) (storage *G*′ (■) and loss *G*″ (▲) modulus) of pure PEO gel and nanocomposite gels obtained at (a) 50 kGy, (b) 130 kGy, (c) 300 kGy from 1.85 wt% PEO suspensions and (d) from 4 wt% PEO suspensions at 130 kGy as a function of time and application of different strains (LVR-DR-LVR) at 25 °C. Linear viscoelastic region (LVR): strain = 0.1%, frequency = 5 Hz; destructive region (DR): strain = 300%, frequency = 5 Hz. Initial mass percentage of Fe^3+^ in precursor suspensions is indicated.
