# Supplementary material for: Rheological, Microstructural and Thermal Properties of Magnetic Poly(Ethylene Oxide)/Iron Oxide Nanocomposite Hydrogels Synthesized Using a One-Step Gamma-Irradiation Method
Source: Nanomaterials (Basel). 2020 Sep 12;10(9):1823. doi: 10.3390/nano10091823 (PMC7559070; doi:10.3390/nano10091823)
Supplement: Supplementary file 1 [file nanomaterials-10-01823-s001.pdf]

Supplementary Materials

Rheological, microstructural and thermal properties of magnetic poly(ethylene oxide)/iron oxide nanocomposite hydrogels synthesized using a one-step gamma-irradiation method

Ivan Marić[1], Nataša Šijaković Vujičić[2], Anđela Pustak[1], Marijan Gotić[3], Goran Štefanić[3], Jean-Marc Grenèche[4], Goran Dražić[5], Tanja Jurkin[1,*]

[1] Radiation Chemistry and Dosimetry Laboratory, Division of Materials Chemistry, Ruđer Bošković Institute, Bijenička cesta 54, 10000 Zagreb, Croatia; imaric@irb.hr (I.M.); apustak@irb.hr (A.P.), tjurkin@irb.hr (T.J.)
[2] Laboratory for Supramolecular Chemistry, Division of Organic Chemistry and Biochemistry, Ruđer Bošković Institute, Bijenička cesta 54, 10000 Zagreb, Croatia; Natasa.Sijakovic-Vujicic@irb.hr (N.Š.V.)
[3] Laboratory for Molecular Physics and Synthesis of New Materials, Division of Materials Physics, Ruđer Bošković Institute, Bijenička cesta 54, 10000 Zagreb, Croatia; gotic@irb.hr (M.G.); Goran.Stefanic@irb.hr (G.Š.)
[4] Institut des Molécules et Matériaux du Mans CNRS UMR-6283, Le Mans Université, Avenue Messiaen, Le Mans F-72085, France; jean-marc.greneche@univ-lemans.fr (J.-M.G.)
[5] Department of Materials Chemistry, National Institute of Chemistry, Hajdrihova 19, SI-1001 Ljubljana, Slovenia; goran.drazic@ki.si (G.D.)
* Correspondence: tjurkin@irb.hr ; Tel.: +385-1-4571-255

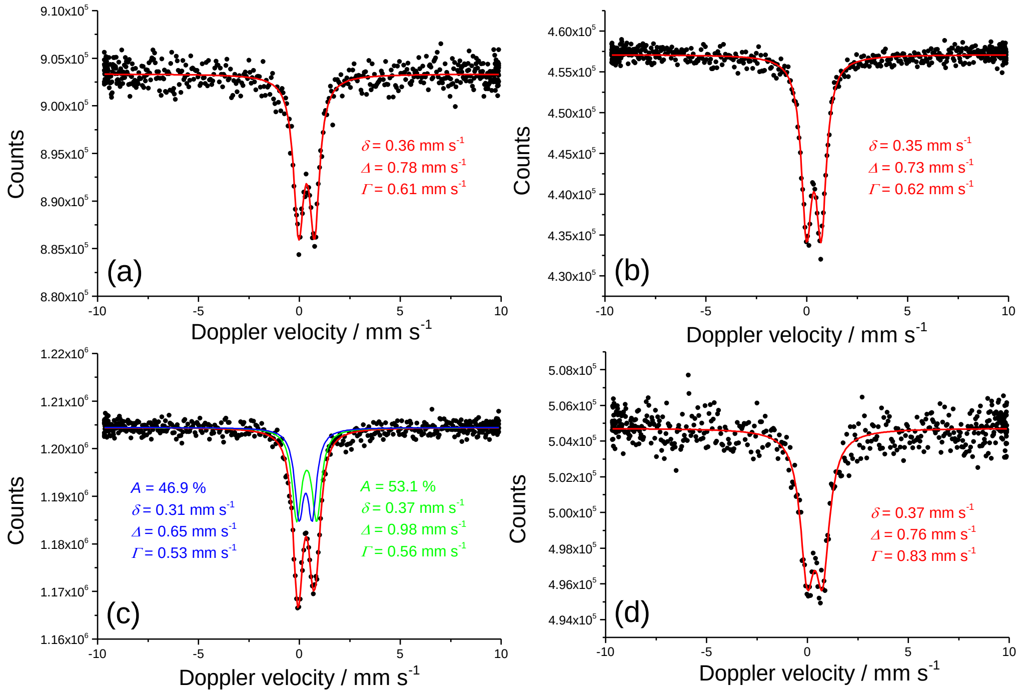

Figure S1. The room temperature Mössbauer spectra of PEO/Fe-oxide gels obtained from suspensions with: (a) 5 wt% Fe^{3+} at 130 kGy; (b) 20 wt% Fe^{3+} at 130 kGy; (c) 5 wt% Fe^{3+} at 300 kGy; (d) 20 wt% Fe^{3+} at 300 kGy. All precursor suspensions were prepared from 1.85 wt% PEO solutions and with 0.2 M 2-propanol. Mössbauer parameters are given: δ = isomer shift relative to α-Fe at 20 °C; Δ = quadrupole splitting; Γ = line width. Error: $\delta = \pm$ 0.01 mm s^{-1}; $\Delta = \pm$ 0.01 mm s^{-1}.

Figure S1 shows the Mössbauer spectra at 20 °C of nanocomposite gels obtained upon irradiation with 2-propanol. All samples were characterized with one Mössbauer doublet having an isomer shift (δ) of about 0.35 – 0.38 mm s^{-1} and a quadrupole splitting (Δ) of about 0.73 – 0.78 mm s^{-1}, except for the Mössbauer spectrum of gel obtained at 300 kGy and 5 wt% initial Fe^{3+} which can be fitted with two doublets. The exact Mössbauer parameters are given on the spectra. The doublets at RT Mössbauerum spectra of all samples can be attributed to superparamagnetic iron oxide particles, but the accurate phase analysis was not possible. Generally, the room temperature Mössbauer spectra depend on the size and crystallinity of iron oxide particles and may vary from a well-shaped sextet like in the case of goethite and maghemite, two sextets in the case of magnetite, down to a doublet characteristic of paramagnetic or very small, superparamagnetic particles [1-5]. Because of the superparamagnetic nature of synthesized nanoparticles and the complex interactions between the superparamagnetic nanoparticles and polymer matrix, the synthesised gels are characterized with broad doublets.

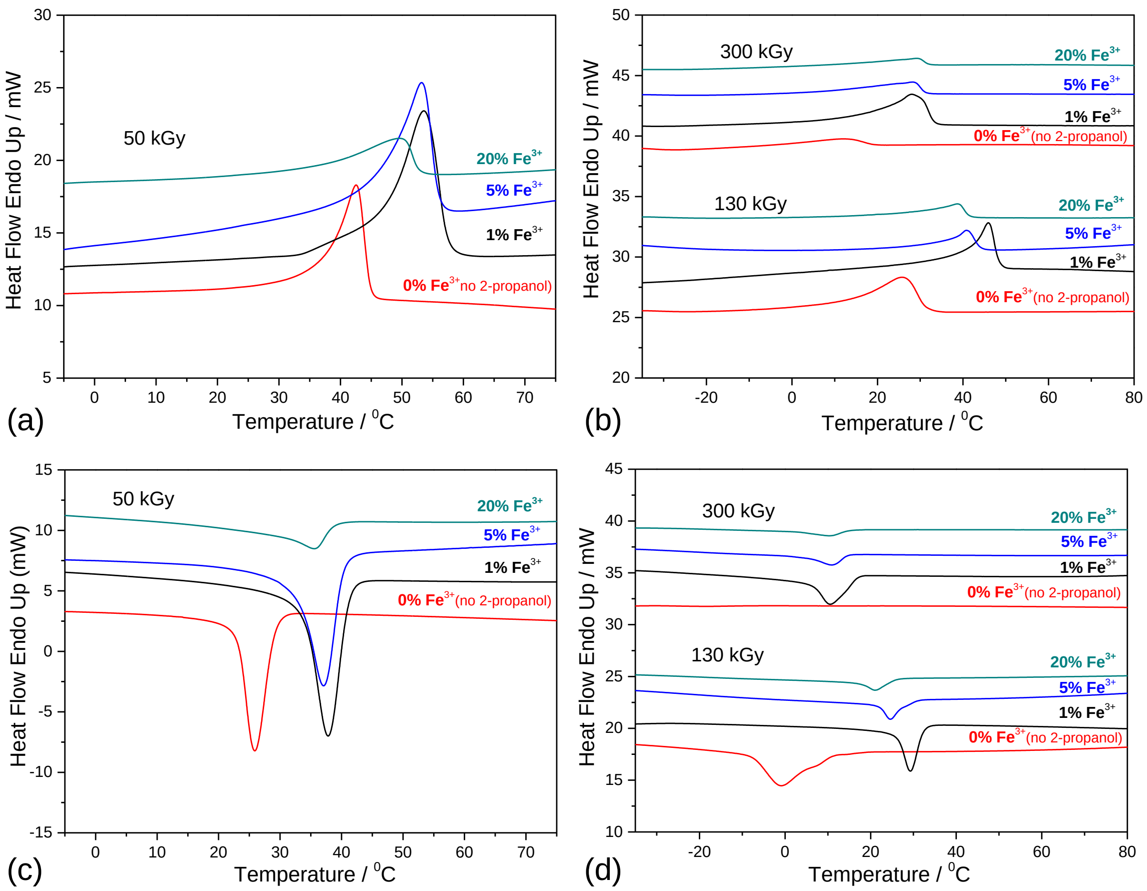

Figure S2. DSC thermographs of the 2nd heating (a,b) and the 1st cooling (c,d) cycles of pure PEO gel and nanocomposite gels obtained at 50, 130 and 300 kGy from 1.85 wt% PEO precursor suspensions with various Fe^{3+} content. Unless otherwise indicated, suspensions contained 0.2 M 2-propanol.

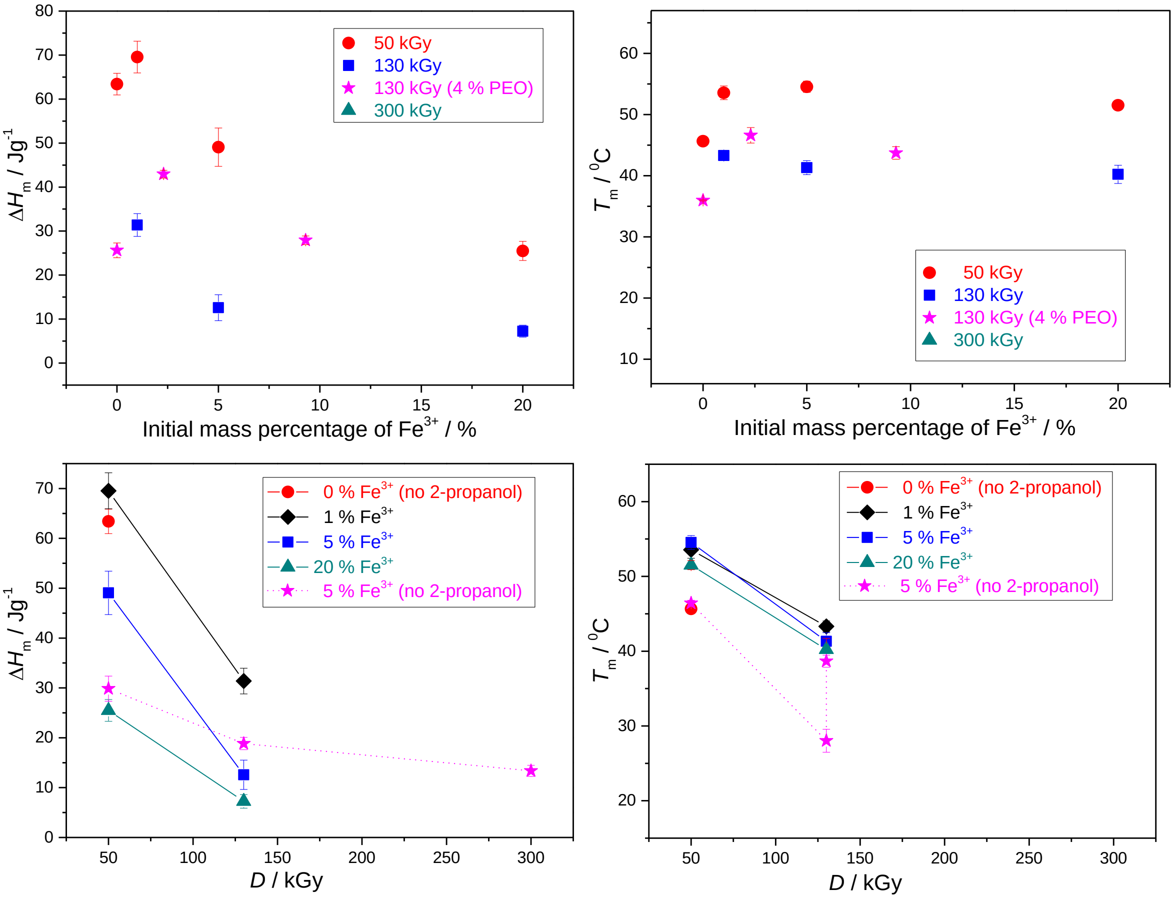

Figure S3. Melting enthalpies and temperatures of the 1st heating cycles of the obtained gels in dependence on the irradiation dose and the mass percentage of Fe^{3+} in precursor suspensions. Unless otherwise indicated, the precursor suspensions were prepared from 1.85 wt% PEO solutions and with addition of 0.2 M 2-propanol. All gels obtained at 300 kGy with 2-propanol, and pure PEO gel at 130 kGy, were totally amorphous in the first heating cycle (no melting enthalpies). Gels obtained by irradiation from 5 wt% Fe^{3+} suspensions without 2-propanol had two melting maxima (both are given on graph).

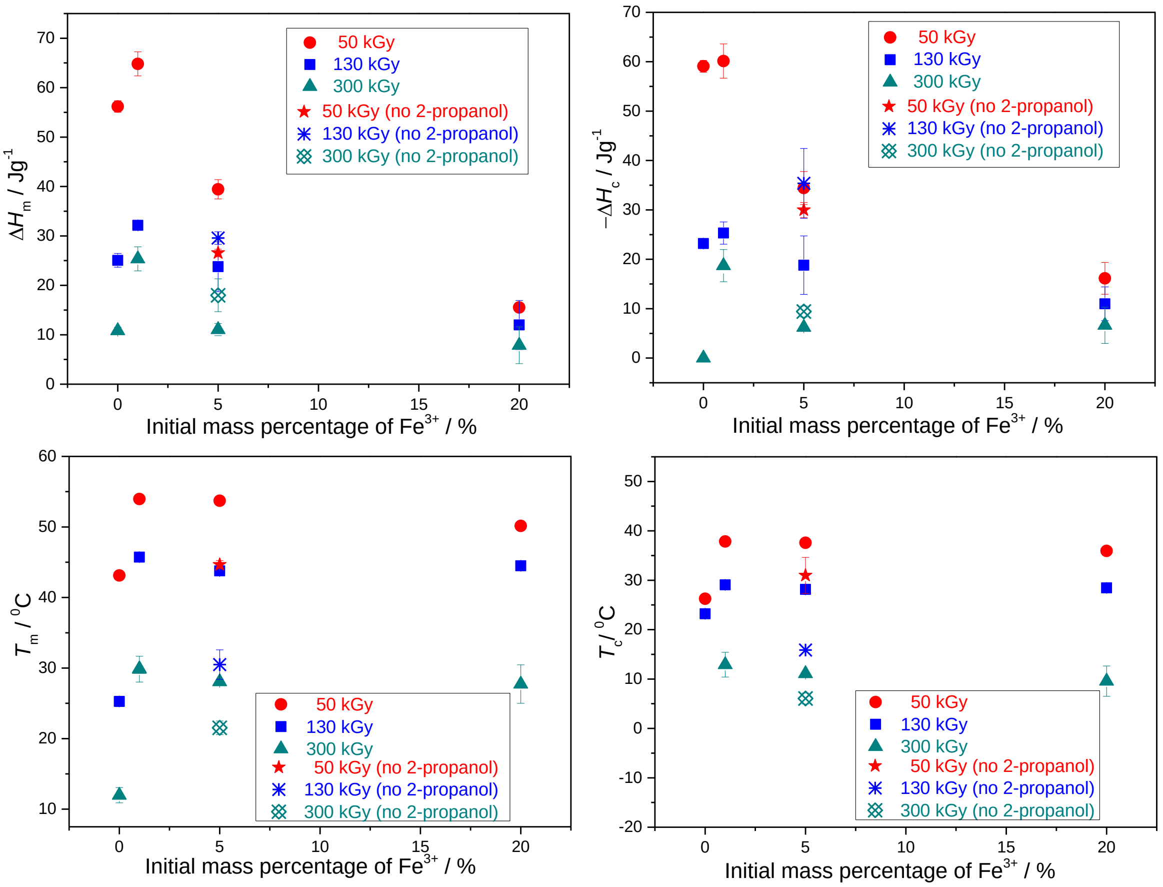

Figure S4. Melting (ΔH_m) and crystallization (ΔH_m) enthalpies and temperatures (T_m and T_c) of the 2nd heating cycles and the 1st cooling cycles, respectively, of gels obtained at various doses in dependence on the mass percentage of Fe^{3+} in 1.85 wt% PEO precursor suspensions. Unless otherwise indicated suspensions contained 0.2 M 2-propanol.

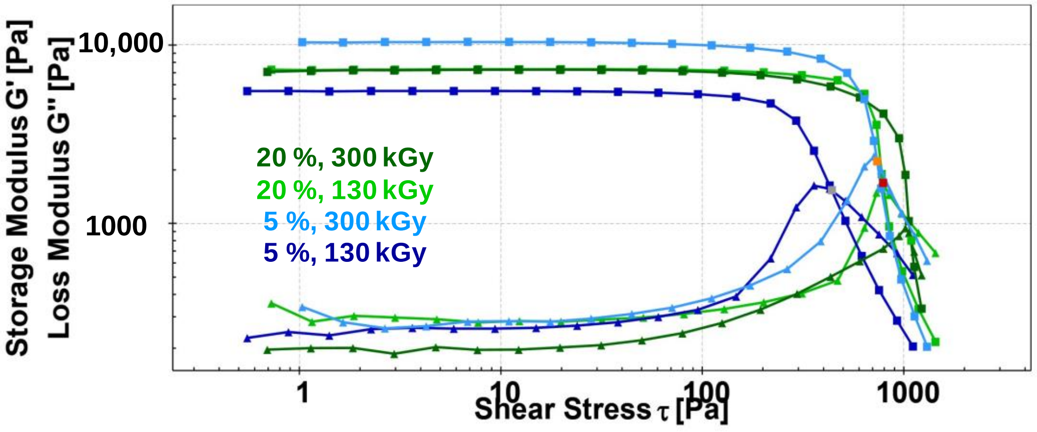

Figure S5. Comparison of amplitude sweep test (G' (■) and G'' (▲) values) of nanocomposite gels obtained at 130 kGy and 300 kGy (1.85 wt% PEO solution), at 25°C. Initial mass percentage of Fe^{3+} in precursor suspensions is indicated.

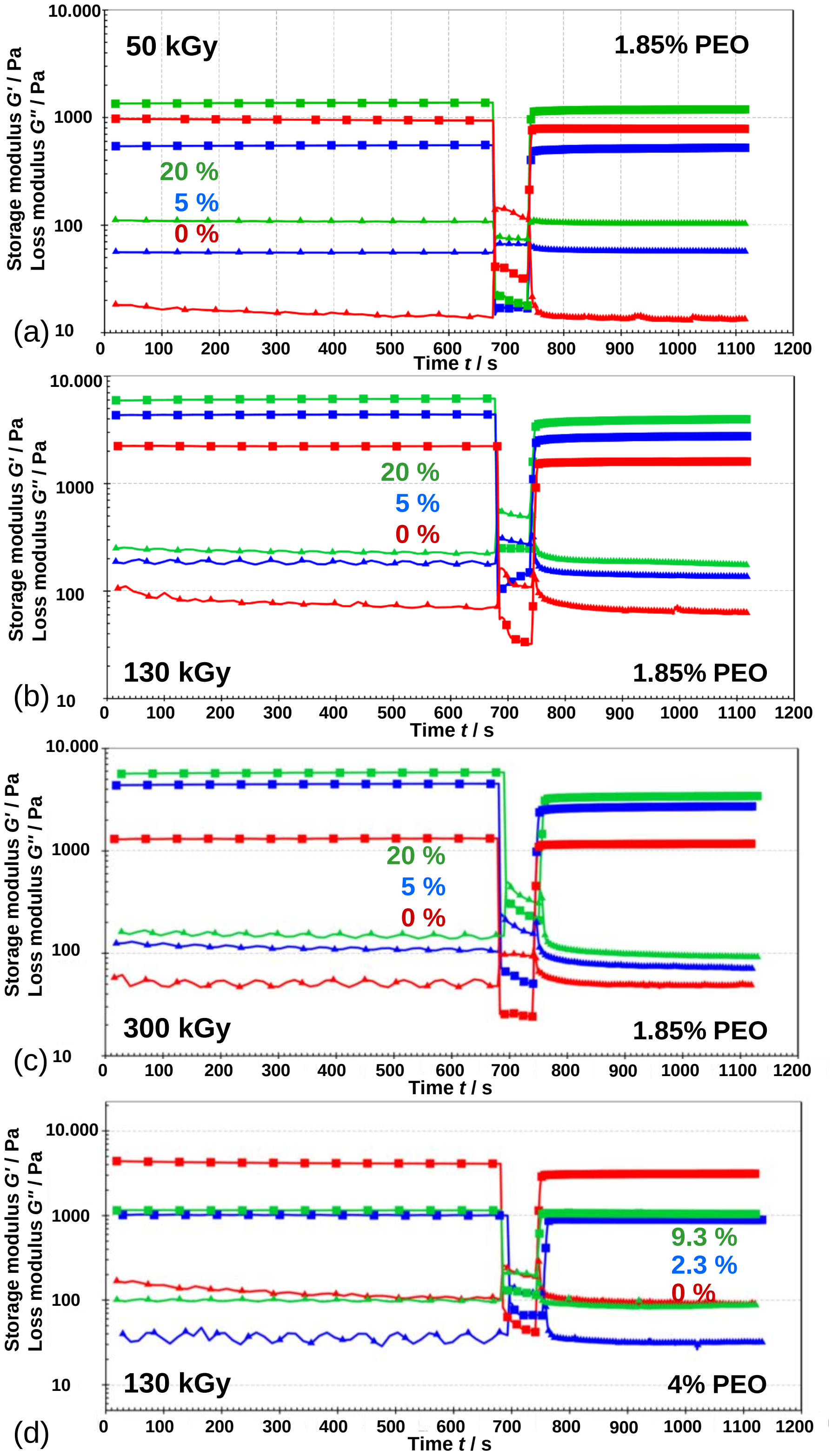

Figure S6. 3-interval thixotropy test (3ITT) (storage G' (■) and loss G'' (▲) modulus) of pure PEO gel and nanocomposite gels obtained at (a) 50 kGy, (b) 130 kGy, (c) 300 kGy from 1.85 wt% PEO suspensions and (d) from 4 wt% PEO suspensions at 130 kGy as a function of time and application of different strains (LVR-DR-LVR) at 25 °C. Linear viscoelastic region (LVR): strain = 0.1 %, frequency = 5 Hz; destructive region (DR): strain = 300 %, frequency = 5 Hz. Initial mass percentage of Fe^{3+} in precursor suspensions is indicated.

References

1. Gotić, M., Jurkin, T.; Musić, S. Factors that may influence the micro-emulsion synthesis of nanosize magnetite particles. *Colloid Polym. Sci.* **2007**, *285*, 793–800. https://doi.org/10.1007/s00396-006-1624-2
2. Gotić, M.; Jurkin, T.; Musić, S. From iron(III) precursor to magnetite and *vice versa*. *Mater. Res. Bull.* **2009**, *44*, 2014–2021. https://doi.org/10.1016/j.materresbull.2009.06.002
3. Gotić, M.; Musić, S. Mössbauer, FT-IR and FE SEM investigation of iron oxides precipitated from $FeSO_4$ solutions. *J. Mol. Struct.* **2007**, *834–836*, 445–453. https://doi.org/10.1016/j.molstruc.2006.10.059
4. Gotić, M., Koščec, G., Musić, S. Study of the reduction and reoxidation of substoichiometric magnetite. *J. Mol. Struct.* **2009**, *924–926*, 347–354. https://doi.org/10.1016/j.molstruc.2008.10.048
5. Gotić, M., Musić, S. Synthesis of nanocrystalline iron oxide particles in the iron(III) acetate/alcohol/acetic acid system. Eur. *J. Inorg. Chem.* **2008**, 966–973. https://doi.org/10.1002/ejic.200700986
